# Supplementary material for: Therapeutic T cells with 3-in-1 strategy for the treatment of biliary tract cancer
Source: Cell Rep Med. 2025 Sep 18;6(10):102349. doi: 10.1016/j.xcrm.2025.102349 (PMC12629817; doi:10.1016/j.xcrm.2025.102349)
Supplement: Document S1. Figures S1–S5 and Tables S1–S10 [file mmc1.pdf]

Cell Reports Medicine, Volume *6*

Supplemental information

Therapeutic T cells with 3-in-1 strategy for the treatment of biliary tract cancer

Xueshuai Wan, Jie Zhao, Xiaobo Yang, Xianing Mou, Bing Liu, Bin Gao, Weiyue Gu, and Haitao Zhao

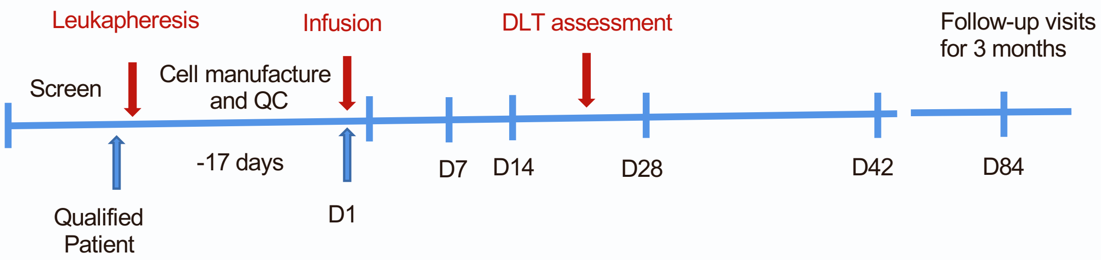

Supplemental Figure S1. Schematic Illustration of the Clinical Study Design. Related to Figure 4,5,6

The figure illustrates the timeline of the clinical trial, encompassing key stages such as screening for qualified patients, leukapheresis, cell manufacturing and quality control (QC), infusion, dose-limiting toxicity (DLT) assessment, and follow-up visits spanning 3 months. The timeline is marked with specific time points: Day 1 (D1) for infusion, and subsequent follow-up assessments on Day 7 (D7), Day 14 (D14), Day 28 (D28), Day 42 (D42), and Day 84 (D84). Leukapheresis is scheduled 17 days prior to infusion.

Supplemental Table S1 HLA typing of Donors. Related to Figure 3A

Donor	HLA typing					
924	HLA-A*02:07	HLA-A*24:02	HLA-B*15:01	HLA-B*46:01	HLA-C*01:02	HLA-C*04:01
678	HLA-A*11:01	HLA-A*30:01	HLA-B*07:02	HLA-B*13:02	HLA-C*06:02	HLA-C*07:02
158	HLA-A*02:07	HLA-A*31:01	HLA-B*40:02	HLA-B*46:01	HLA-C*01:02	HLA-C*04:01

Supplemental Table S2 Candidate neoantigen peptides. Related to Figure 3A

Donor	Gene	Amino acid change	Mutation type	Peptide name	Peptide Seq.	HLA	%Rank_EL(Affinity) #
924	IER5	p.L15V	Missense	924-1-IER5	VSISVGKIY	HLA-B*46:01	0.124
	PLSCR2	p.L238F	Missense	924-2-PLSCR2	SFDEQIVV	HLA-C*04:01	0.036
				924-3-PLSCR2	GVDFEITSF	HLA-C*04:01	0.449
158	XIRP2	p.L1869P	Missense	158-1-XIRP2	NMPATLKSL	HLA-A*02:07	0.431
	TAOK3	p.Q722Tfs*14	Frameshift	158-2-TAOK3	MEMQIKKTV	HLA-B*40:02	0.059
	MADD	p.Y1285C	Missense	158-3-MADD	RVYLCEGLLGR	HLA-A*31:01	0.11
	ATP6V0A4	p.S611R	Missense	158-4-ATP6V0A4	HAPRILIHF	HLA-B*46:01	0.288
	OXER1	p.I275S	Missense	158-5-OXER1	IVSSGLTIRNR	HLA-A*31:01	0.345
	MGA	p.A2372V	Missense	158-6-MGA-1	TTAVHTQSF	HLA-B*46:01	0.107
				158-7-MGA-2	VAHLKTTAV	HLA-C*01:02	0.443
	MGA	p.H2373Qfs*32	Frameshift	158-8-MGAfs-1	AAQTHSHSNSR	HLA-A*31:01	0.469
				158-9-MGAfs-2	VERLHQFL	HLA-B*40:02	0.475
				158-10-MGAfs-3	NSRPVLTSL	HLA-B*46:01	0.281
678	SACS	p.L3395V	Missense	678-1-SACS	YFNCNVNHL	HLA-C*07:02	0.364
	DCSTAMP	p.R439W	Missense	678-2-DCSTAMP	RWLSLYLTK	HLA-A*30:01	0.266
	LOC729159	p.P99Pfs*22	Frameshift	678-3-LOC-1	VVWSPPRGR	HLA-A*11:01	0.312
				678-4-LOC-2	WSPPRGRNL	HLA-C*06:02	0.363
	SRRM2	p.Q1828Qfs*36	Frameshift	678-5-SRRM2-1	RQFPDLLSTPK	HLA-A*11:01	0.276
				678-6-SRRM2-2	HSRSPARQFPDLL	HLA-B*07:02	0.076
				678-7-SRRM2-3	TPNQSEAFSLTHI	HLA-B*07:02	0.256
	CCT8L2	p.C72Vfs*30	Frameshift	678-8-CCT8L2-1	SSMAPPGSR	HLA-A*30:01	0.39
				678-9-CCT8L2-2	HPQGPGAGAPSSM	HLA-B*07:02	0.146
	CUBN	p.F832Ffs*68	Frameshift	678-10-CUBN-1	RLVAVPFWVLLK	HLA-A*11:01	0.043
				678-11-CUBN-2	KIKSIAVQTYL	HLA-A*30:01	0.374
				678-12-CUBN-3	KAKSFSSTSLSLK	HLA-A*30:01	0.45
				678-13-CUBN-4	LPTVKQIMLRLV	HLA-B*07:02	0.211
				678-14-CUBN-5	IRSPFFLTCIL	HLA-B*07:02	0.413
				678-15-CUBN-6	STSPKAKSF	HLA-C*06:02	0.308
	NEURL	p.D29Dfs*107	Frameshift	678-16-NEURL1-1	APRGHPQNL	HLA-B*07:02	0.002
				678-17-NEURL1-2	SPMPPQAEAL	HLA-B*07:02	0.009
				678-18-NEURL1-3	APSHAAAL	HLA-B*07:02	0.079
				678-19-NEURL1-4	YRGPLPRHFSPM	HLA-B*07:02	0.117
				678-20-NEURL1-5	GPHLRASQA	HLA-B*07:02	0.483
				678-21-NEURL1-6	LQQPPGPHL	HLA-B*13:02	0.078
				678-22-NEURL1-7	GQLLQRHHL	HLA-B*13:02	0.138
				678-23-NEURL1-8	SQAEDHQEAV	HLA-B*13:02	0.338

*, The mutated amino acids are marked in bold, italic and underlined.
#, Rank threshold for strong binding peptides: <0.500.
From NetMHCpan-4.1,https://services.healthtech.dtu.dk/services/NetMHCpan-4.1/

Supplemental Table S3. Demographics of patients who received ScTIL cells therapy. Related to Figure 5 and Table 1

Characteristic	Statistic	Low (N=4) 1.0×10^8	Medium (N=3) 5.0×10^8	High (N=3) 1.0×10^9	Total (N=10)
Age	N	4	3	3	10
	Mean(SD)	58(9.83)	57.67(7.23)	62(4)	59.1(7.17)
	Median	59	54	62	58.5
	Min, Max	46, 70	53, 66	58, 66	46,70
	Q1~Q3	54.25, 61.75	53.5, 60	60, 64	54.75, 65
Sex	All	4	3	3	10
	Male (%)	3(75.0)	2(66.7)	2(66.7)	7(70.0)
	Female (%)	1(25.0)	1(33.3)	1(33.3)	3(30.0)
Nationality	All	4	3	3	10
	Han Nationality (%)	3(75.0）	3(100)	3(100)	9(90.0)
	Others (%)	1(25.0)	0	0	1(10.0)
Baseline Weight (Kg)	N(missing)	3(1)	3(0)	3(0)	9(1)
	Mean(SD)	76.5(16.26)	67.5(7.50)	53.5(24.06)	66.09(13.25)
	Median	78.8	67.5	57.5	65
	Min, Max	65,88	60,75	43,60	43,88
	Q1~Q3	70.75, 82.25	63.75, 71.25	50.3, 58.8	60, 75

Supplemental Table S4 Treatment emergent adverse events, TEAE. Related to Figure 5

AE term	Frequency	Incidence (N=10)	Rate
Metabolic and Nutritional Diseases	14	6	60
Hypoalbuminemia	2	2	20
Hyponatremia	1	1	10
Loss of appetite	2	2	20
Hyperuricemia	4	2	20
Hypertriglyceridemia	1	1	10
Hyperglycemia	2	2	20
Hypernatremia	1	1	10
Hyperkalemia	1	1	10
Systemic diseases and various reactions at the administration site	9	6	60
Weak	2	1	10
Fever	7	6	60
Various tests	46	6	60
Elevated C-reactive protein	1	1	10
Elevated alanine aminotransferase	3	1	10
High neutrophil count	1	1	10
Low neutrophil count	4	2	20
Weight loss	1	1	10
Prolonged prothrombin time	1	1	10
Elevated aspartate aminotransferase	2	2	20
Increased anti-transglutaminase antibodies	1	1	10
Prolonged activated partial thromboplastin time	1	1	10
Low lymphocyte count	3	3	30
High white blood cell count	1	1	10
Low white blood cell count	3	1	10
Elevated fibrin D-dimer	1	1	10
Elevated conjugated bilirubin	5	3	30
Low platelet count	4	2	20
Elevated alkaline phosphatase	4	3	30
Elevated creatinine	3	2	20
Elevated bilirubin	7	4	40
Various nervous system diseases	3	3	30
Dizziness	1	1	10
Headache	2	2	20
Respiratory, thoracic and mediastinal diseases	2	1	10
Respiratory failure	1	1	10
Hypoxia	1	1	10
Infections and infestations	2	2	20
Upper respiratory infection	1	1	10
Abdominal wall abscess	1	1	10
Skin and Subcutaneous Tissue diseases	1	1	10
Rash	1	1	10
Mental illness	1	1	10
Insomnia	1	1	10
Gastrointestinal Diseases	6	5	50
lower gastrointestinal bleeding	1	1	10
constipate	1	1	10
nausea	1	1	10
stomach ache	1	1	10
bloating	1	1	10
noninfectious gingivitis	1	1	10
Blood and Lymphatic System Diseases	3	3	30
Anemia	3	3	30

Supplemental Table S5 Treatment related adverse events, TRAE. Related to Figure 5

AE term	Frequency	Incidence (out of 10)	Rate
Metabolic and Nutritional Diseases	2	2	20
Loss of appetite	2	2	20
Systemic diseases and various reactions at the administration site	7	6	60
Fever	7	6	60
Blood tests	13	5	50
Elevated alanine aminotransferase	3	1	10
Decreased neutrophil count	1	1	10
Decreased lymphocyte count	2	2	20
Elevated conjugated bilirubin	2	1	10
Low platelet count	1	1	10
Elevated alkaline phosphatase	2	2	20
Elevated bilirubin	2	1	10
Nervous system diseases	2	2	20
Headache	2	2	20
Mental Illness	1	1	10
Insomnia	1	1	10
Gastrointestinal Diseases	3	3	30
Constipation	1	1	10
Nausea	1	1	10
Bloating	1	1	10

Supplemental Table S6 Treatment related adverse events (TRAE) by grade. Related to Figure 5

Adverse event	Incidence N (%) *				Frequency M (%) **			
(N=10)	Cumulative Cases	Grade 1	Grade 2	>= Grade 3	Cumulative frequency	Grade 1	Grade 2	>=Grade 3
All	8(80.0)	2(20.0)	3(30.0)	3(30.0)	28(100.0)	15(53.6)	8(28.6)	5(17.9)
Fever	6(60.0)	4(40.0)	2(20.0)	0(0.0)	7(25.0)	5(17.9)	2(7.1)	0(0.0)
Loss of appetite	2(20.0)	2(20.0)	0(0.0)	0(0.0)	2(7.1)	2(7.1)	0(0.0)	0(0.0)
Headache	2(20.0)	2(20.0)	0(0.0)	0(0.0)	2(7.1)	2(7.1)	0(0.0)	0(0.0)
Decreased Lymphocyte count	2(20.0)	0(0.0)	0(0.0)	2(20.0)	2(7.1)	0(0.0)	0(0.0)	2(7.1)
Elevated bilirubin	1(10.0)	0(0.0)	1(10.0)	0(0.0)	2(7.1)	1(3.6)	1(3.6)	0(0.0)
Elevated alkaline phosphatase	1(10.0)	0(0.0)	0(0.0)	1(10.0)	2(7.1)	0(0.0)	1(3.6)	1(3.6)
Low platelet count	1(10.0)	0(0.0)	1(10.0)	0(0.0)	1(3.6)	0(0.0)	1(3.6)	0(0.0)
constipate	1(10.0)	1(10.0)	0(0.0)	0(0.0)	1(3.6)	1(3.6)	0(0.0)	0(0.0)
nausea	1(10.0)	1(10.0)	0(0.0)	0(0.0)	1(3.6)	1(3.6)	0(0.0)	0(0.0)
bloating	1(10.0)	0(0.0)	1(10.0)	0(0.0)	1(3.6)	0(0.0)	1(3.6)	0(0.0)
Elevated alanine aminotransferase	1(10.0)	0(0.0)	1(10.0)	0(0.0)	3(10.7)	2(7.1)	1(3.6)	0(0.0)
Decreased neutrophil count	1(10.0)	0(0.0)	1(10.0)	0(0.0)	1(3.6)	0(0.0)	1(3.6)	0(0.0)
Insomnia	1(10.0)	1(10.0)	0(0.0)	0(0.0)	1(3.6)	1(3.6)	0(0.0)	0(0.0)
elevated conjugated bilirubin	1(10.0)	0(0.0)	0(0.0)	1(10.0)	2(7.1)	0(0.0)	0(0.0)	2(7.1)

*The denominator is all subjects who have used the drug at least once, and each subject only selects the most severe level for calculation.

** The denominator is the frequency of all AEs, and the numerator is the frequency of each grade.

Supplemental Table S7. OS, PFS and DCR analysis for treated subjects in different dosage groups or in Low & Middle-dose, and High-dose groups. A. OS and PFS analysis for treated subjects in 1.0×10^8, 5.0×10^8, 1.0×10^9 groups. Related to Figure 5; B. DCR results of treated subjects in 1.0×10^8, 5.0×10^8, and 1.0×10^9 dose groups; C. OS and PFS analysis for treated subjects in Low& Middle-dose, and High-dose groups; D. DCR results of treated subjects in Low & Middle-dose, and High-dose groups. Related to Figure 5.

S7A OS and PFS analysis for treated subjects in 1.0×10^8, 5.0×10^8, 1.0×10^9 groups. Related to Figure 5.

Indicator	Statistics	1.0×10^8 N=4	5.0×10^8 N=3	1.0×10^9 N=3	Total N=10	P value
OS(Month)	Death	3(75.0)	3(100)	3(100)	9(90.0)	
	Censored	1(25.0)	0	0	1(10.0)	
	Median	14.6	18.3	3.2	8.4	0.107
	95%CI	(0.16, 23.56)	(8.38, 19.84)	(2.56,4.01)	(0.16,19.84)	
PFS(Month)	PD/Death	3(75.0)	3(100)	3(100)	9(90.0)	
	Censored	1(25.0)	0	0	1(10.0)	
	Median	3.39	6.83	1.35	1.48	0.085
	95%CI	(0.16,5.42)	(2.73,12.02)	(1.15,1.48)	(0.16, 6.83)	

Censored：One patient of censored in OS is S006, who was lost to follow-up after being discharged;

One patient of censored in PFS is S010, who underwent subsequent treatment immediately after the initial evaluation for SD.

S7B. DCR results of treated subjects in 1.0×10^8, 5.0×10^8, and 1.0×10^9 dose groups. Related to Figure 5.

Indicator	Statistics	1.0×10^8 N=4	5.0×10^8 N=3	1.0×10^9 N=3	Total N=10
DCR-6W	N(Missing)	3(1)	3(0)	3(0)	9(1)
	DCR(%)	2(50.0)	3(100)	0	5(50.0)
DCR-12W	N(Missing)	1(3)	3(0)	0(3)	4(6)
	DCR(%)	1(25.0)	2(66.7)	0	3(30.0)

Missing：S004 died on day 5 after cell infusion, and no efficacy evaluation results were obtained at weeks 6 and 12; S006, S010, S024, S025, and S029 were withdrawn from the study before the 12-week assessment.

S7C. OS and PFS analysis for treated subjects in Low& Middle-dose, and High-dose groups. Related to Figure 5.

Indicator	Statistics	Low&Middle-dose (1~5E8) N=7	High-dose (1E9) N=3	Total N=10	P Value
OS(Month)	Death	6(85.7)	3(100.0)	9(90.0)	
	Censored	1(14.3)	0	1(10.0)	
	Median	18.3	3.15	8.38	0.035
	95%CI	(0.16,23.56)	(2.56,4.01)	(0.16,19.84)	
PFS(Month)	PD/Death	6(85.7)	3(100.0)	9(90.0)	
	Censored	1(14.3)	0	1(10.0)	
	Median	5.42	1.35	1.48	0.081
	95%CI	(0.16,12.02)	(1.15,1.48)	(0.16, 6.83)	

Censored：One patient of censored in OS is S006, who was lost to follow-up after being discharged;

One patient of censored in PFS is S010, who underwent subsequent treatment immediately after the initial evaluation for SD.

S7D. DCR results of treated subjects in Low&Middle-dose, and High-dose groups. Related to Figure 5.

Indicator	Statistics	Low&Middle-dose (1~5E8) N=7	High-dose (1E9) N=3	Total N=10	P Value
OS(Month)	Death	6(85.7)	3(100.0)	9(90.0)	
	Censored	1(14.3)	0	1(10.0)	
	Median	18.3	3.15	8.38	0.035
	95%CI	(0.16,23.56)	(2.56,4.01)	(0.16,19.84)	
PFS(Month)	PD/Death	6(85.7)	3(100.0)	9(90.0)	
	Censored	1(14.3)	0	1(10.0)	
	Median	5.42	1.35	1.48	0.081
	95%CI	(0.16,12.02)	(1.15,1.48)	(0.16, 6.83)	

Missing：S004 died on day 5 after cell infusion, and no efficacy evaluation results were obtained at weeks 6 and 12; S006, S010, S024, S025, and S029 were withdrawn from the study before the 12-week assessment.

Supplemental Table S8. OS, PFS and DCR analysis for treated subjects. A OS and PFS analysis for treated subjects with B cell normal and B cell low of B cells groups; B. DCR results of treated subjects with B cell normal and B cell low of B cells; C. Additional OS and PFS analysis for treated subjects with B%N50 and B%L50 levels of B cells groups; D. Additional DCR results of treated subjects with B%N50 and B%L50 levels of B cells. Related to Figure 5.

S8A. OS and PFS analysis for treated subjects with B cell normal and B cell low of B cells groups. Related to Figure 5.

Indicator	Statistics	B cell normal N=3	B cell low N=7	Total N=10	P Value
OS(Month)	Death	3(100)	6(85.7)	9(90.0)	
	Censored	0	1(14.3)	1(10.0)	
	Median	18.3	4.01	8.38	0.409
	95%CI	(14.62,19.84)	(0.16,23.56)	(0.16,19.84)	
PFS(Month)	PD/Death	3(100)	6(85.7)	9(90.0)	
	Censored	0	1(14.3)	1(10.0)	
	Median	6.83	1.35	1.48	0.015
	95%CI	(5.42,12.02)	(0.16,2.73)	(0.16, 6.83)	

Censored：One patient of censored in OS is S006, who was lost to follow-up after being discharged;

One patient of censored in PFS is S010, who underwent subsequent treatment immediately after the initial evaluation for SD.

S8B. DCR results of treated subjects with B cell normal and B cell low of B cells. Related to Figure 5.

Indicator	Statistics	B cell normal N=3	B cell low N=7	Total N=10
DCR-6W	N(Missing)	3(0)	6(1)	9(1)
	DCR(%)	3(100)	2(28.6)	5(50.0)
DCR-12W	N(Missing)	3(0)	1(6)	4(6)
	DCR(%)	3(100)	0	3(30.0)

Missing：S004 died on day 5 after cell infusion, and no efficacy evaluation results were obtained at weeks 6 and 12; S006, S010, S024, S025, and S029 were withdrawn from the study before the 12-week assessment.

S8C. Additional OS and PFS analysis for treated subjects with B%N50 and B%L50 levels of B cells groups. Related to Figure 5.

Indicator	Statistics	B%N50 N=7	B%L50 N=3	Total N=10	P Value
OS(Month)	Death	7(100.0)	2(66.7)	9(90.0)	
	Censored	0	1(33.3)	1(10.0)	
	Median	14.62	3.15	8.38	0.031
	95%CI	(2.56,19.84)	(0.16,3.15)	(0.16,19.84)	
PFS(Month)	PD/Death	6(85.7)	3(100.0)	9(90.0)	
	Censored	1(14.3)	0	1(10.0)	
	Median	5.42	1.35	1.48	0.056
	95%CI	(1.15,12.02)	(0.16,1.48)	(0.16, 6.83)	

B% N50 : Baseline B-cell proportion ≥50% LLN；B% L50 : Baseline B-cell proportion ＜50%LLN
Censored：One patient of censored in OS is S006, who was lost to follow-up after being discharged;
One patient of censored in PFS is S010, who underwent subsequent treatment immediately after the initial evaluation for SD.

S8D. Additional DCR results of treated subjects with B%N50 and B%L50 levels of B cells. Related to Figure 5.

Indicator	Statistics	B%N50 N=7	B%L50 N=3	Total N=10
DCR-6W	N(Missing)	7(0)	2(1)	9(1)
	DCR(%)	5(71.4)	0	5(50.0)
DCR-12W	N(Missing)	4(3)	0(3)	4(6)
	DCR(%)	3(42.9)	0	3(30.0)

B% N50 : Baseline B-cell proportion ≥50% LLN；B% L50 : Baseline B-cell proportion ＜50%LLN
Missing：S004 died on day 5 after cell infusion, and no efficacy evaluation results were obtained at weeks 6 and 12; S006, S010, S024, S025, and S029 were withdrawn from the study before the 12-week assessment.

Supplemental Table S9. A. OS , PFS and DCR analysis for combined factor of B%50 & 1-5 x 10^8 and control dose groups. A. OS and PFS analysis for combined factor of B%50 & 1-5 x 10^8 and control dose groups; B. DCR analysis for combined factor of B%50 &1-5x10^8 dose groups Related to Figure 5.

S9A. OS and PFS analysis for combined factor of B%50 & 1-5 x 10^8 and control dose groups, Related to Figure 5.

Indicator	Statistics	B%50&1~5E8 N=5	Control N=5	Total N=10	P Value
OS(Month)	Death	5(100)	4(80.0)	9(90.0)	0.003
	Censored	0	1(20.0)	1(10.0)	
	Median	18.3	3.15	8.38	
	95%CI	(8.38,23.56)	(0.16,4.01)	(0.16,19.84)	
PFS(Month)	PD/Death	4(80.0)	5(100)	9(90.0)	0.003
	Censored	1(20.0)	0	1(10.0)	
	Median	6.13	1.35	1.48	
	95%CI	(2.73,12.02)	(0.16,1.48)	(0.16, 6.83)	

B%50&1~5x 10^8: Baseline B-cell proportion ≥50% LLN with dose of 1~5x10^8
Censored：One patient of censored in OS is S006, who was lost to follow-up after being discharged; One patient of censored in PFS is S010, who underwent subsequent treatment immediately after the initial evaluation for SD.

S9B. DCR analysis for combined factor of B%50 &1-5x10^8 dose groups. Related to Figure 5.

Indicator	Statistics	B%50&1~5x 10^8 N=5	Control N=5	Total N=10
DCR-6W	N(Missing)	5(0)	4(1)	9(1)
	DCR(%)	5(100)	0	5(50.0)
DCR-12W	N(Missing)	4(1)	0(5)	4(6)
	DCR(%)	3(60.0)	0	3(30.0)

B%50&1~5x10^8: Baseline B-cell proportion ≥50% LLN with dose of 1~5x10^8
Missing：S004 died on day 5 after cell infusion, and no efficacy evaluation results were obtained at weeks 6 and 12; S006, S010, S024, S025, and S029 were withdrawn from the study before the 12-week assessment.

Supplemental Table S10. Proportion of $CD19^+$ B cells (%) to leukocytes at Baseline, D7, D14, D28, D42 and D84. Related to Figure 4A.

Groups	Patient	Baseline	Days After Infusion				
			D7	D14	D28	D42	D84
1.0×10^8	S003	12.89	5.1	0	0	1	9.7
	S004	2.30	-	-	-	-	-
	S006	3.5	4.2	0.1	0.5	0.5	-
	S010	5.4	2.7	0.3	0.4	1.2	-
5.0×10^8	S014	11	8.5	4.4	4.4	5.2	7.6
	S016	11.1	6.4	4.6	4.9	4.7	7.1
	S023	7.5	6.9	0.3	0.6	1.4	7.8
1.0×10^9	S024	8.4	0.6	2.5	2.7	0.8	-
	S025	6	0.1	0	0.4	0.4	-
	S029	2	-	0	0.3	0.1	-
TOTAL	N(Missing)	10(0)	8(2)	8(2)	8(2)	8(2)	4(6)
	Mean	7.01	4.31	1.36	1.58	1.70	8.05
	SD	3.84	3.01	1.95	1.91	1.89	1.14
	Median	6.75	4.65	0.30	0.50	1	7.70
	MAX	12.89	8.50	4.60	4.90	5.20	9.70
	MIN	2	0.10	0	0	0.10	7.10

Supplemental Figure S2. Kaplan-Meier analysis of the progression-free survival and overall survival between different combination groups. A. Kaplan-Meier analysis of the progression-free survival and overall survival between different dosage groups; B. Survival analysis for subjects in combined dose groups; C. Survival analysis for subjects in different baseline B%; D. Survival analysis for subjects in baseline B%≥ or < 50%LLN.

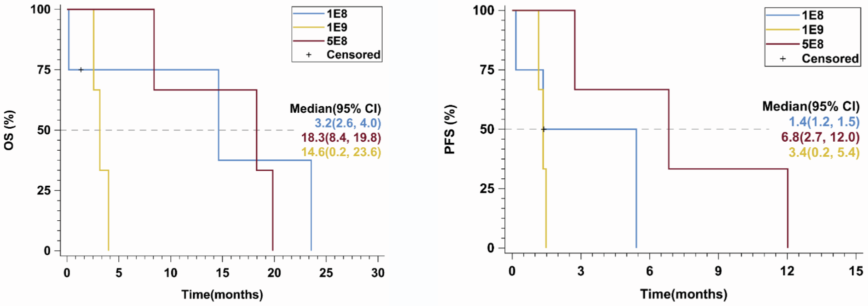

S2A. Kaplan-Meier analysis of the progression-free survival and overall survival between different groups. Left Panel: Overall Survival, OS. Right Panel: Progression-free Survival, PFS. Related to Fig. 5D.

Left Plot (Overall Survival, OS): Lines: Represent survival curves for subjects in different dose groups. - Blue line: Dose group of 1E8. Median OS [95% Confidence Interval (CI)] is 3.2 months (2.6, 4.0). - Yellow line: Dose group of 1E9. Median OS [95% CI] is 18.3 months (8.4, 19.8). - Red line: Dose group of 5E8. Median OS [95% CI] is 14.6 months (0.2, 23.6).
"+" Markers: Indicate censored data points (subjects who did not experience the event of interest, e.g., death, during the study follow - up).

Right Plot (Progression - Free Survival, PFS): - Lines: Represent progression - free survival curves for subjects in different dose groups. - Blue line: Dose group of 1E8. Median PFS [95% CI] is 1.4 months (1.2, 1.5). - Yellow line: Dose group of 1E9. Median PFS [95% CI] is 6.8 months (2.7, 12.0). - Red line: Dose group of 5E8. Median PFS [95% CI] is 3.4 months (0.2, 5.4).
"+" Markers: Indicate censored data points (subjects who did not have disease progression during the study follow - up).

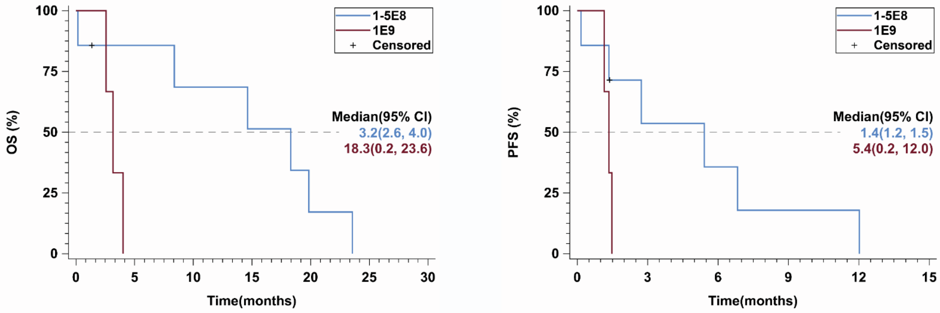

S2B Survival analysis for subjects in combined dose groups. Left Plot: Overall Survival, OS. Right Plot: Progression-Free Survival, PFS. Related to Fig 5D.

Left Plot (Overall Survival, OS) : Lines: - Blue line: Combined dose group "1 – 5x10^8". Median OS [95% Confidence Interval (CI)] is 3.2 months (2.6, 4.0). - Red line: Dose group "1x10^9". Median OS [95% CI] is 18.3 months (0.2, 23.6).

Right Plot (Progression - Free Survival, PFS): Lines: Blue line: Combined dose group "1 – 5x10^8". Median PFS [95% CI] is 1.4 months (1.2, 1.5). Red line: Dose group "1x10^9". Median PFS [95% CI] is 5.4 months (0.2, 12.0).

"+" Markers: Indicate censored data (subjects without disease progression during follow - up).

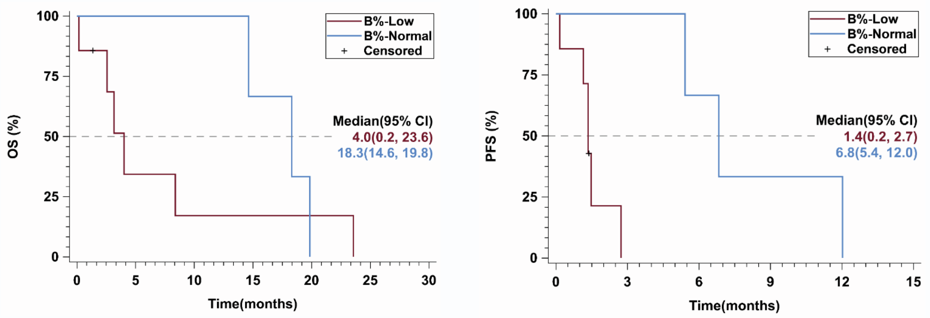

S2C Survival analysis for subjects in different baseline B%. Left Plot: Overall Survival, OS. Right Plot: Progression-Free Survival, PFS. Related to Figure 5D

Left Plot (Overall Survival, OS): Lines: Red line: Group with low baseline B% (B%-Low). Median OS [95% Confidence Interval (CI)] is 4.0 months (0.2, 23.6). Blue line: Group with normal baseline B% (B%-Normal). Median OS [95% CI] is 18.3 months (14.6, 19.8).

Right Plot (Progression-Free Survival, PFS): Lines: Red line: Group with low baseline B% (B%-Low). Median PFS [95% CI] is 1.4 months (0.2, 2.7). Blue line: Group with normal baseline B% (B%-Normal). Median PFS [95% CI] is 6.8 months (5.4, 12.0).

"+" Markers: Indicate censored data points (subjects who did not have disease progression during the study follow-up

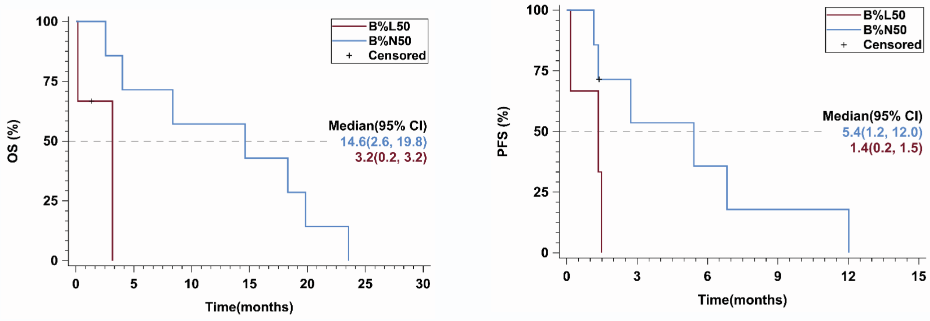

S2D Survival analysis for subjects in baseline B%≥ or < 50%LLN. Left Plot: Overall Survival, OS. Right Plot; Progression-Free Survival, PFS. Related Figure 5D.

Left Plot (Overall Survival, OS): Lines: Red line: Group with baseline B% < 50% of the lower limit of normal (B%L50). Median OS [95% Confidence Interval (CI)] is 3.2 months (0.2, 3.2). -Blue line: Group with baseline B% ≥ 50% of the lower limit of normal (B%N50). Median OS [95% CI] is 14.6 months (2.6, 19.8).

Right Plot (Progression-Free Survival, PFS) : Lines: Red line: Group with baseline B% < 50% of the lower limit of normal (B%L50). Median PFS [95% CI] is 1.4 months (0.2, 1.5). Blue line: Group with baseline B% ≥ 50% of the lower limit of normal (B%N50). Median PFS [95% CI] is 5.4 months (1.2, 12.0).

"+" Markers: Indicate censored data points (subjects who did not have disease progression during the study follow-up).

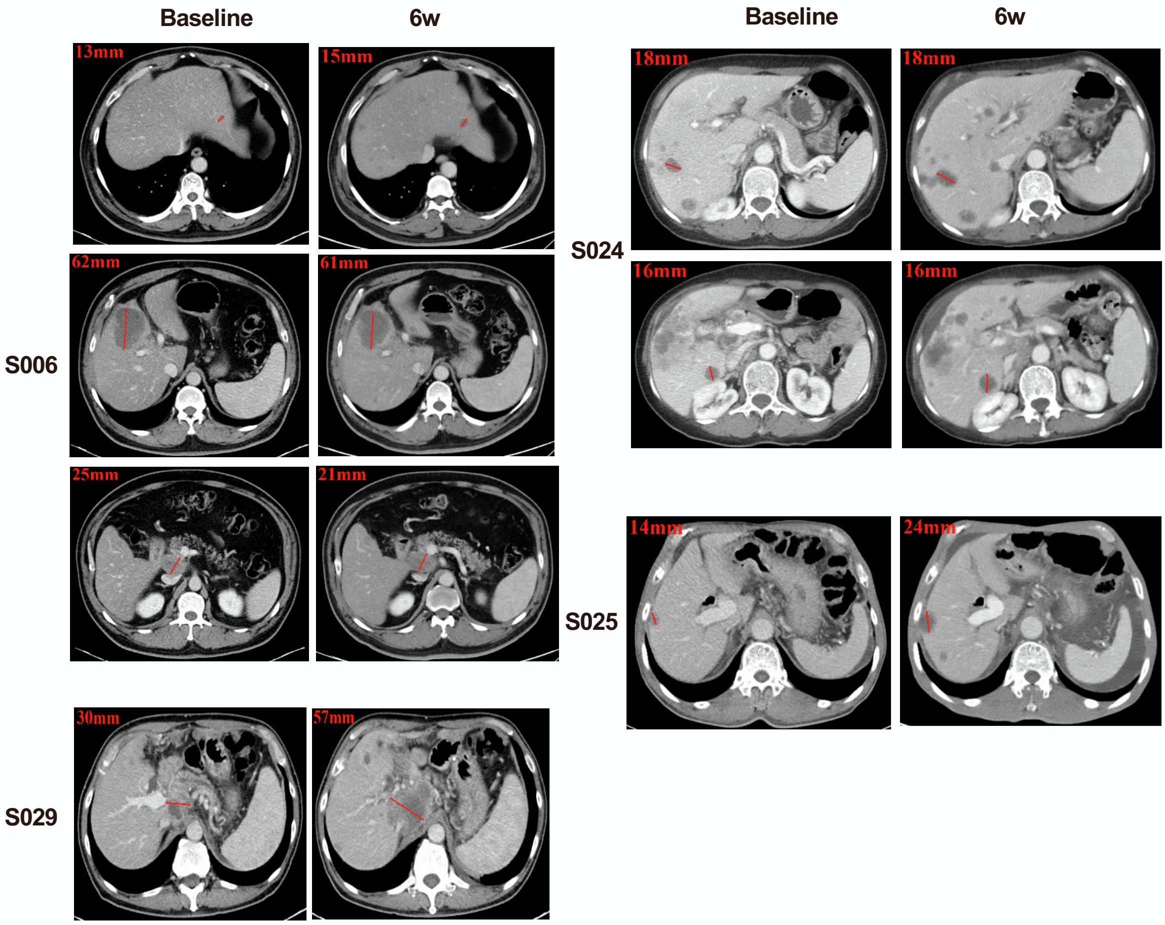

Supplemental Figure S3. Target lesions' CT imaging of baseline and different evaluation time points in progressive disease patients. CT images of patients (S006, S024, S025, S029) with low baseline B cells at baseline and 6 weeks post-treatment are presented. **Related Figure 5A.**

Computed tomography (CT) images of patients (S006, S024, S025, S029) with low baseline B cells at baseline and 6 weeks (6w) post-treatment are presented. For S006, one lymph node (upper panel) and two liver lesions (middle and lower panel) are shown; for S024, two liver lesions; for S025, one liver lesion; and for S029, one lymph node, all at baseline and 6 weeks post-treatment. Lesion sizes (in milli-meter) are indicated in red, and red arrows highlight target lesions. S006 and S024 showed stable disease with no significant change in lesion size. In contrast, S025 and S029 showed an increase in lesion size at 6 weeks compared to baseline.

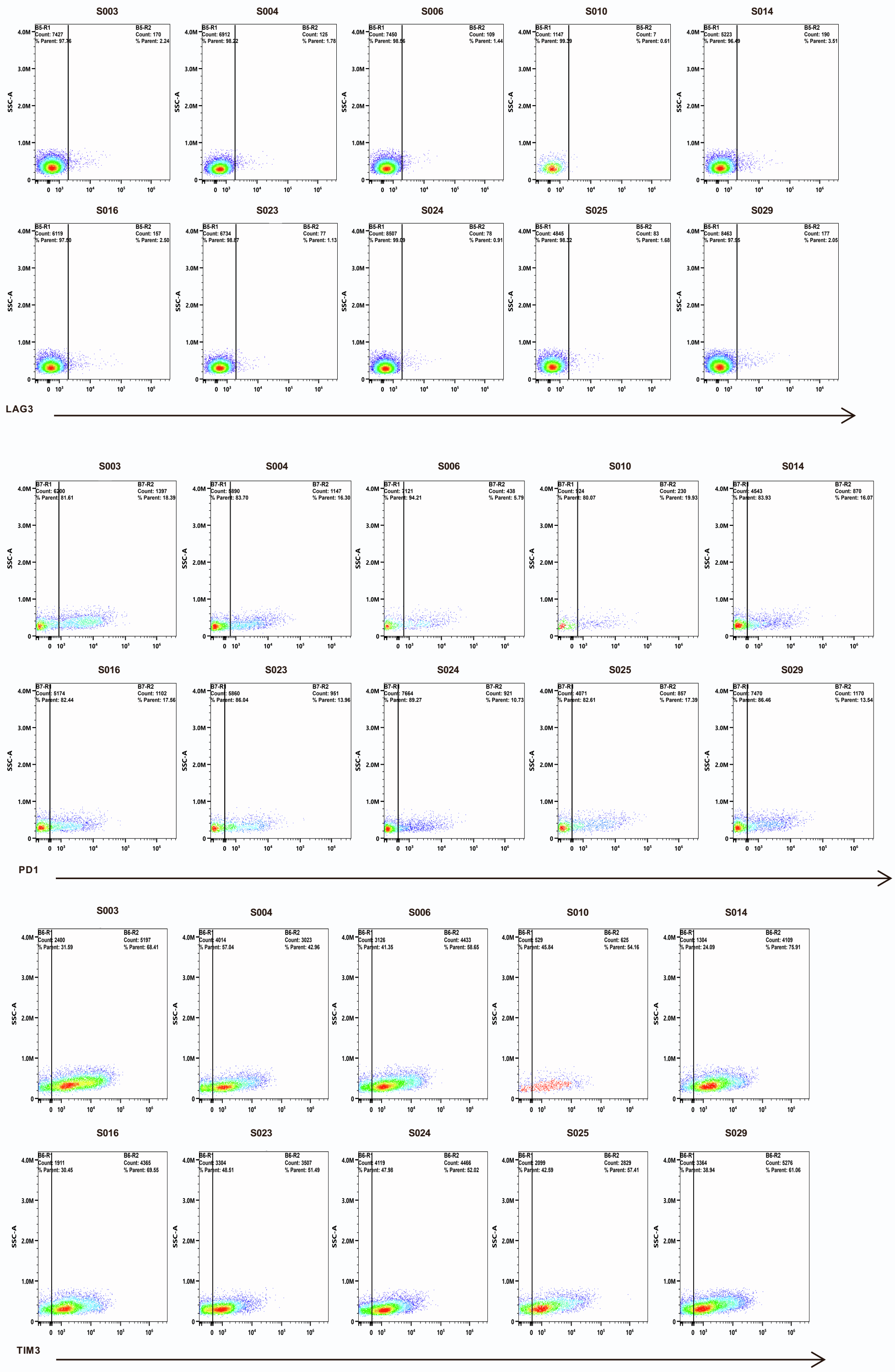

Supplemental Figure S4. Analysis of percentages of PD1, LAG3 and TIM3T in cell products from different patients

T cell products from different patients are stained with commercial antibodies against PD1, LAG3 and TIM3 respectively and analysed with flow cytometry. Related Figure 4.

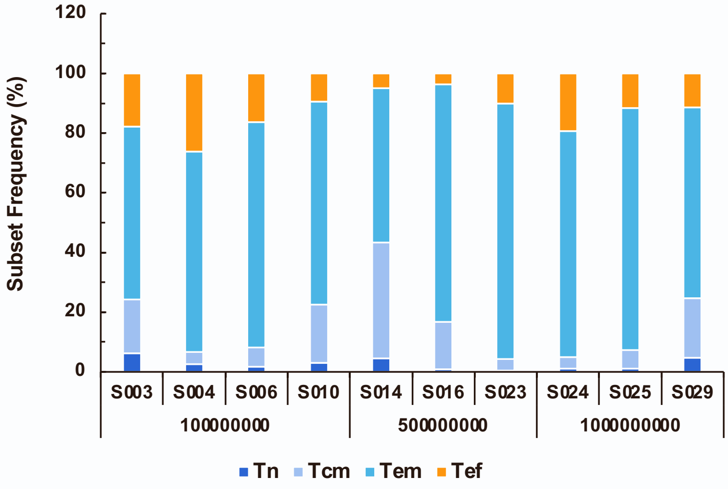

Supplemental Figure S5. Memory phenotypes of different dosages of T cell products from different patients. Related Figure 4 and 5.

Memory phenotypes are characterized by surface staining of CD45RA and CD62L and analysed with flow cytometry (Tn: CD45RA+/ CD62L+; Tcm: CD45RA-/ CD62L+; Tem: CD45RA-/ CD62L- and Tef: CD45RA+/ CD62L-).
