# Supplementary material for: Therapeutic T cells with 3-in-1 strategy for the treatment of biliary tract cancer
Source: Cell Rep Med. 2025 Sep 18;6(10):102349. doi: 10.1016/j.xcrm.2025.102349 (PMC12629817; doi:10.1016/j.xcrm.2025.102349)
Supplement: Document S2. Article plus supplemental information [file mmc2.pdf]

Cell Reports
Medicine

Article

Therapeutic T cells with 3-in-1 strategy for the treatment of biliary tract cancer

Graphical abstract

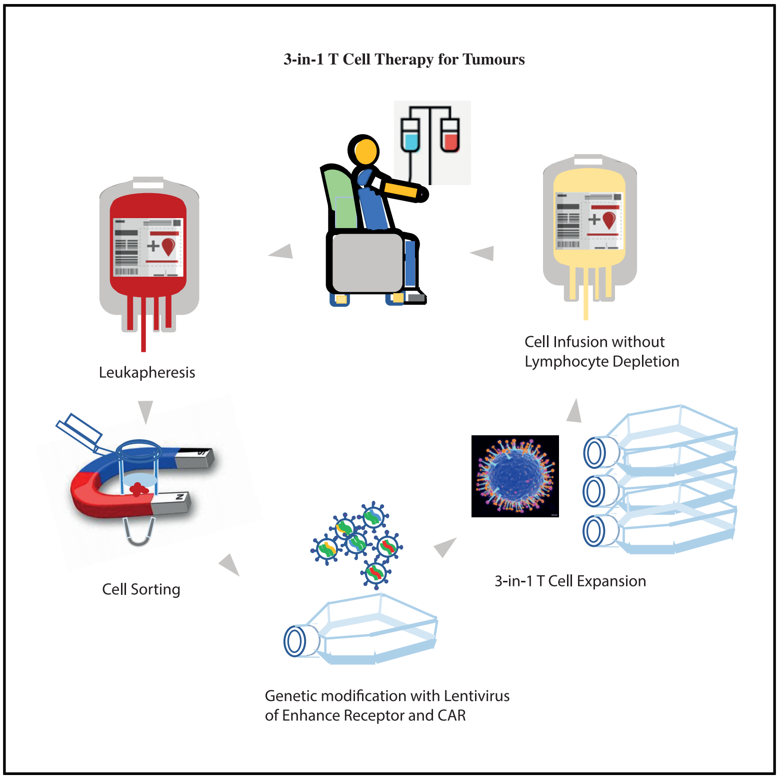

Authors

Xueshuai Wan, Jie Zhao, Xiaobo Yang, ..., Bin Gao, Weiyue Gu, Haitao Zhao

Correspondence

bgao2004@icloud.com (B.G.),
guweiyue@cartmed.org (W.G.),
zhaoht@pumch.cn (H.Z.)

In brief

Wan et al. develop a 3-in-1 strategy for T cell therapy that demonstrates effective treatment outcomes for advanced biliary tract cancer (BTC). In a selected patient cohort, this approach achieved a median overall survival (OS) of 18.3 months, outperforming available standard therapy.

Highlights

- 3-in-1 ScTILs for PD-1+ T cells modified with ER, PD-1/CD28, and anti-CD19 CAR
- ScTILs effectively kill tumor cells *in vitro* and *in vivo* with strong antitumor activity
- In selected BTC, ScTILs outperform standard therapy with18.3-month median OS
- ScTILs skip chemo-pre-treatment/IL-2 without surgical materials and is safer and quicker

Wan et al., 2025, Cell Reports Medicine *6*, 102349
October 21, 2025 © 2025 The Authors. Published by Elsevier Inc.
https://doi.org/10.1016/j.xcrm.2025.102349

Cell Reports Medicine

CellPress
OPEN ACCESS

Article

Therapeutic T cells with 3-in-1 strategy for the treatment of biliary tract cancer

Xueshuai Wan,[1,6] Jie Zhao,[2,6] Xiaobo Yang,[1,6] Xianing Mou,[3] Bing Liu,[4] Bin Gao,[5,7,*] Weiyue Gu,[5,*] and Haitao Zhao[1,*]
[1]Chinese Academy of Medical Sciences & Peking Union Medical College, Beijing, China
[2]Preclinical Research and Development, Chineo Medical Technology Co. Ltd, Beijing, China
[3]Gene and Cell Therapy Process Development, Chineo Medical Technology Co. Ltd, Beijing, China
[4]Clinical Medicine, Chineo Medical Technology Co. Ltd, Beijing, China
[5]Chineo Medical Technology Co. Ltd, Beijing, China
[6]These authors contributed equally
[7]Lead contact
*Correspondence: bgao2004@icloud.com (B.G.), guweiyue@cartmed.org (W.G.), zhaoht@pumch.cn (H.Z.)
https://doi.org/10.1016/j.xcrm.2025.102349

SUMMARY

T cell therapy for tumors faces barriers like heterogeneous antigen expression, an unfriendly tumor microenvironment, and limited T cell expansion. We adopt a 3-in-1 strategy to produce super circulating TIL-like (tumor-infiltrating lymphocyte-like) cells (ScTILs): modifying PD-1-positive peripheral blood T cells with an enhance receptor (ER), a PD-1 and CD28 fusion protein to reverse inhibitory signal, and an anti-CD19 chimeric antigen receptor (CAR) for expansion (CFE). ScTILs kill tumor cells effectively *in vitro* and *in vivo*. Clinically, ten advanced biliary tract cancer (BTC) patients receive ScTILs treatment; *post hoc* analysis shows that ScTILs monotherapy yields a median overall survival (OS) of 18.3 months in appropriate dose or normal B cell groups (5/10), outperforming first-line BTC treatment (OS ∼12 months). It skips chemo-pre-treatment and interleukin-2 (IL-2), with better safety, no reliance on surgical materials, and a shorter production cycle. Overall, ScTILs are a promising therapy for future BTC treatment. This study is registered with ChiCTR (ChiCTR2000029738).

INTRODUCTION

Chimeric antigen receptor (CAR) T's clinical success in hematological malignancies has reversed the view of cancer as incurable. Buoyed by this, cell therapy was expected to breakthrough in solid tumors. However, solid tumor clinical trials[1] show CAR-T, while relatively safe, has disappointing efficacy compared to its success in hematological cancers. Additionally, alternative strategies like T cell receptor (TCR)-T, natural killer, and other solid tumor-targeted cellular therapies have not achieved major breakthroughs. T cell therapy for solid tumors faces three key barriers: (1) heterogeneous antigens, (2) tumor microenvironment (TME) inhibiting T cell function, and (3) difficulty producing sufficient non-exhausted T cells *in vitro*.

Solid tumors are highly heterogeneous,[2,3] so effector cells targeting a single antigen cannot address their diverse antigens; that is why CAR-T fails here as it works in hematological cancers. Tumors comprise multiple cell lineages with distinct somatic mutations[4]; targeted therapies (e.g., inhibitors and CAR-T/TCR-T) only hit discrete neoantigens, often generated via Darwinian selection over time. Even with full clearance of dominant cancer cells, residual escaped cells can proliferate. Thus, broader T cell target coverage is needed. To combat heterogeneity, two proven techniques exist: expanding tumor-infiltrating lymphocytes (TILs) and developing neoantigen-specific T cells. TILs, effective at tumor sites, have multi-targeted TCR poly-clonalities, superior homing, and low toxicity, outperforming other approaches.[5] Yet TILs manufacture is laborious (reliant on scarce surgical material and skilled techniques); they show poor viability/long-term survival, especially after prolonged culture *in vitro*. Autologous TILs *in vitro* culture has low success rates, with preparation issues leading to patient dropout. Moreover, TILs treatment requires pre-chemotherapy plus high-dose interleukin-2 (IL-2) (high side effects), and efficacy is limited to few tumors (e.g., melanoma and cervical cancer).[6]

Given TILs limitations, focus shifted to peripheral blood cells for multi-targeted, tumor-reactive lymphocytes. As early as 1990, Itoh's lab found that, in 12 renal cell carcinoma patients, peripheral blood T lymphocyte (PBL) clones had formation rates similar to/higher than TILs in half the cases, with PBL cytotoxicity correlating with TILs[7]; most PBL clones lysing autologous tumors also targeted allogeneic antigens, matching TILs cytotoxicity. Rosenberg's team later identified PD-1 expression on peripheral blood lymphocytes as a marker for neoantigen-targeting T cells: TILs antigen-specific CD8 T cells highly expressed PD-1[8]; they isolated neoantigen-specific T cells from peripheral

© 2025 The Authors. Published by Elsevier Inc.
This is an open access article under the CC BY license (http://creativecommons.org/licenses/by/4.0/).

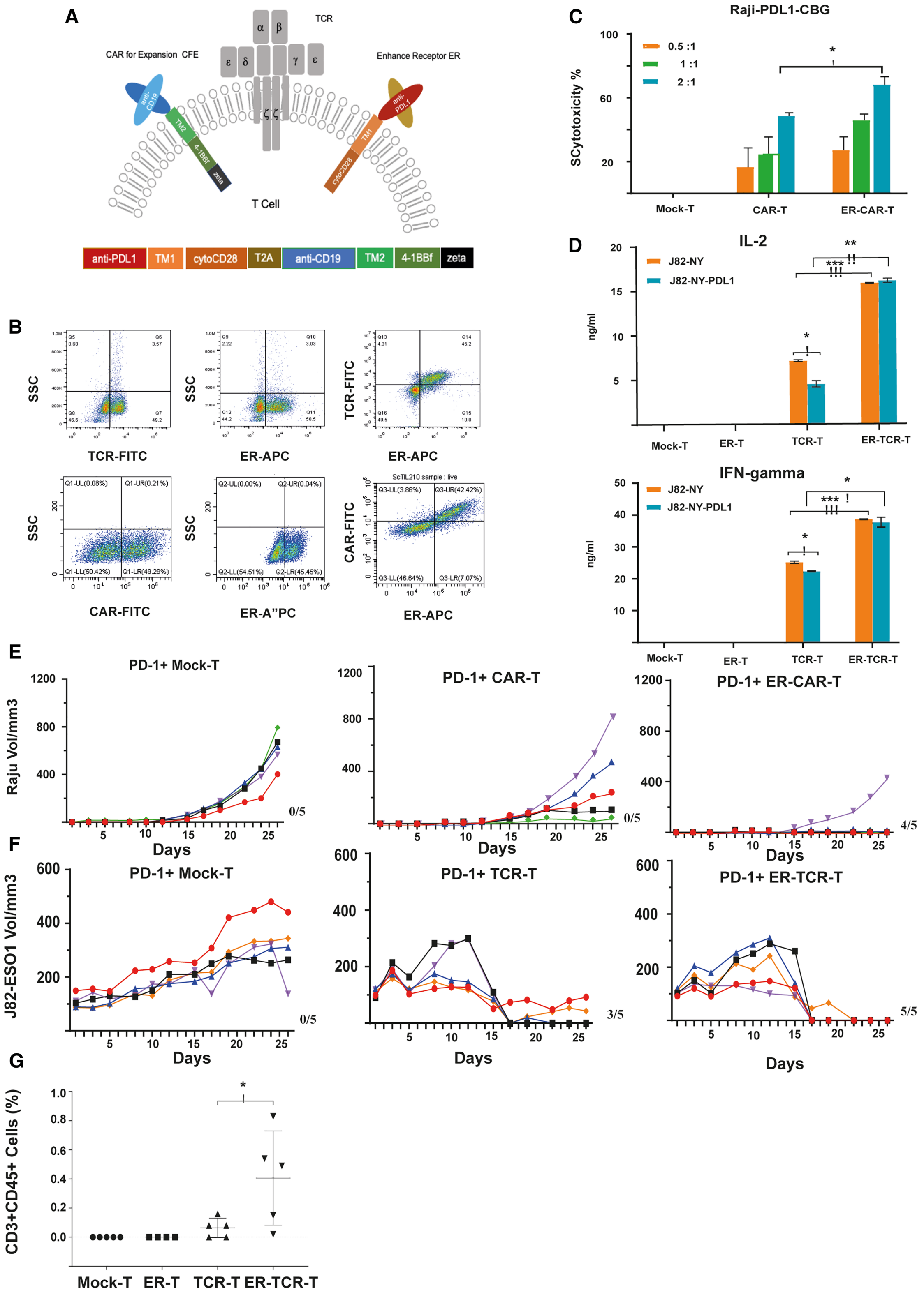

(legend on next page)

blood PD-1+ lymphocytes in 3/4 melanoma patients via high-throughput sequencing, showing these cells target neoantigens.[9] They further found that peripheral blood PD-1+ T cells share specificity and TCR composition with tumor-infiltrating T cells,[10] and personalized neoantigen-specific T cells could be enriched from gastrointestinal cancer patients' blood using PD-1, with their TCRs recognizing autologous tumors. Liu et al. noted most antigen-recognizing T cells are PD-1+.[11] Gao's lab confirmed that peripheral blood tumor-specific T cells mainly express PD-1; combining these T cells with anti-PD-1 antibodies yielded 1 complete response (CR), 1 partial response (PR), 1 stable disease (SD) in melanoma patients and 1 PR in a kidney cancer patient.[12] Thus, peripheral blood PD-1+ T cells in cancer patients, like TILs, recognize heterogeneous tumors and are termed circulating TIL-like cells (cTILs).

The TME's immunosuppressive molecules inhibiting T cell function are the second barrier. Solid tumors have a hostile TME with regulation of immune checkpoints. Strategies to overcome this include anti-PD-1 antibodies,[13,14] disrupting PD-1 in CAR-T via CRISPR-Cas9,[15] or expressing dominant-negative PD-1 extracellular domains on CAR-T.[16] These restore suppressed T cell function[14] and show promise in early trials.[17–20] Another strategy uses "enhance receptors" (ERs or "switch molecules"),[21,22] fusion proteins with a PD-L1 receptor extracellular segment (e.g., PD-1), and intracellular co-stimulatory domain (e.g., CD28), to reverse PD-L1's inhibitory signal into T cell activation.

Therapeutic T cells need extensive expansion to match tumor cell numbers, but large-scale expansion of non-senescent T cells remains problematic. The rapid expansion protocol using allogeneic leukocytes[23] enables massive expansion *in vitro* but requires allogeneic cells, with resource and safety challenges. In hematological CAR-T, infused cells can expand *in vivo* via B cell activation; Ghassemi et al. found omitting *in vitro* expansion and infusing T cells earlier allow *in vivo* expansion via B cell stimulation, yielding "younger," more effective cells.[24] This suggests CD19 CAR could act as an *in vivo* "expansion factor" for solid tumor patients with normal B cells.

To address these three barriers, we developed a 3-in-1 strategy for therapeutic T cells (ScTILs, super cTILs): (1) isolating PD-1+ T cells (cTILs) from patients' peripheral blood, which recognize heterogeneous tumors like TILs; (2) modifying cTILs with an ER (PD-L1 receptor plus CD28 activating fragment) to reverse TME inhibition; and (3) co-expressing an expansion factor (CAR for expansion [CFE]), an anti-CD19 CAR, enabling ScTILs to expand rapidly *in vivo* via B cells to sufficient numbers. ScTILs used to treat biliary tract malignancy significantly extended advanced patients' survival, raising hopes for solid tumor treatment.

RESULTS

ScTILs kill tumor cells *in vitro* and suppress tumor growth in xenotransplant animal models

We sorted out PD-1-positive T cells in the periphery as cTILs and modified them with the following expression cassettes by lentiviral transfection as shown in Figure 1A. (1) A fusion protein called the enhance receptor (ER) fusing single-chain variable fragment (scFv) of anti-PD-L1 to the intracellular domain of CD28 molecule. In the presence of PD-L1, the fusion protein provides activation signal to stimulate T cells to counteract the inhibitory effect of PD-L1 in the TME. (2) The CAR structure targeting the B cell antigen CD19 (CFE) co-expressed by Thosea asigna virus 2A peptide (T2A) in the same framework as the enhance receptor. Substantial expansion of transduced T cells would happen after transfusion back through stimulation provided by CD19 antigen on B cells in blood.)

PD-1-positive T cells stimulated by CD3/CD28 magnetic beads were transduced with lentiviruses co-expressing ER and CFE and cultured in serum-free X-VIVO 15 media with IL-2 and

Figure 1. Structure of ScTILs with native TCR and enhance receptor improved the function of T cells

(A) The schematic diagram illustrates the structure of ScTILs, which includes the native TCR, an enhance receptor (ER) made up of an anti-PD-L1 scFv fused with a transmembrane region and the cytoplasmic fragment of CD28 molecule, and a CAR for expansion (CFE) that consists of scFv to CD19 fused to a transmembrane region, cytoplasmic fragment of 4-1BB, and the epsilon subunit of the CD3 complex.

(B) Expression of ER and TCR or ER and CFE on cell surface demonstrated by flow cytometry analysis. Flow cytometry confirmed the expression of ER and TCR on the surface of ER-TCR-T cells and the expression of ER and CFE on the ER-CAR-T cells. The top three figures depict the detection of both ER and TCR on ER-TCR-T cells while the lower three figures show the expression of ER and CAR on ER-CAR T cells. In both cases, the ratio of double-positive cells was found to be above 40%.

(C) Comparative cytotoxicity analysis of MOCK-T, CAR-T, and ER-CAR-T cells against Raji-PD-L1-CBG cells at different T:E ratios. The cytotoxicity levels of MOCK-T, CAR-T, and ER-CAR-T cells against Raji-PD-L1-CBG cells were analyzed at T:E ratios of 0.5:1, 1:1, and 2:1, respectively. The results show a comparative analysis of the cytotoxicity levels achieved by the three different cells. The p values less than 0.05, less than 0.01, and less than 0.001 are represented by *, **, and ***, respectively.

(D) Analysis of cytokine release from ER-TCR-T cells co-cultured with J82- NY or J82-NY-PD-L1 target cells. The ELISA assay was used to analyze the levels of IFN-γ and IL-2 released by ER-TCR-T cells when co-cultured with J82-NY or J82-NY-PD-L1 target cells. The analysis was done based on the supplier's kit instructions, and the results represent the average (mean ± SD) obtained from three independent experiments. The p values less than 0.05, less than 0.01, and less than 0.001 are represented by *, **, and ***, respectively.

(E) Antitumor effect of MOCK-T, CAR-T, and ER-CAR-T cells on Raji tumor models. Mice inoculated with Raji tumor cells subcutaneously were intravenously infused with MOCK-T, CAR-T, or ER-CAR-T cells prepared from peripheral blood PD-1+ T cells after 30 min. The tumor volume was measured at different time points, and the results are presented as mean tumor volume (±SEM) for five mice per group. The numbers beside the horizontal axis show the number of mice with a complete response (CR) out of five mice in each group.

(F) Evaluation of the antitumor effect of ER-TCR-T cells on J82-NY xenograft model. NSG mice were subcutaneously inoculated with J82-NY- GFP tumor cells. When the average tumor volume reached 100 mm^3, mice were randomly split into groups and infused with MOCK-T, TCR-T, or ER- TCR-T cells. Tumor volume was measured at different time points, and the results are presented as mean tumor volume (±SEM) for five mice per group. The numbers beside the horizontal axis indicate the number of mice demonstrating a complete response (CR) out of five mice in each group.

(G) T cells survived in tumor tissues in different treatments of animals.

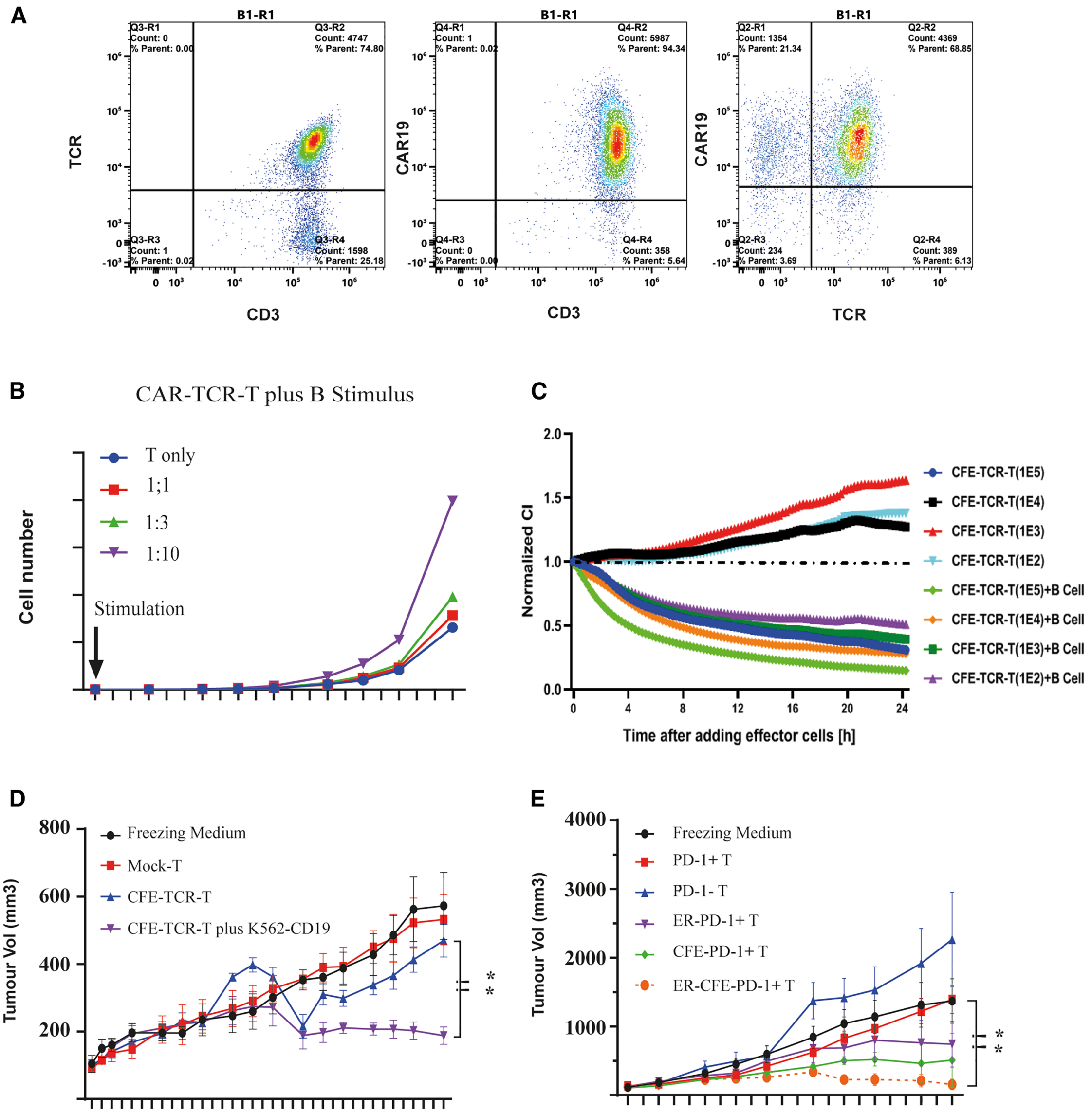

Figure 2. CFE expanded ScTILs in the presence of B cells and effectively suppressed tumors growth *in vivo*

(A) Flow cytometry confirms the expression of CFE and TCR on the surface of CFE-TCR-T cells. The left figure depicts the detection of TCR on CD3-positive cells, and the middle figure show the expression of CFE on CD3-positive cells. In right figure, CFE and TCR are simultaneously expressed on about 65% of T cells.

(B) Effect of B cell stimulation on the expansion of CFE-TCR-T cells. The expansion of CFE-TCR-T cells was monitored under B cell stimulation. CFE- TCR-T cells were mixed with B cells (T:B) in varying ratios of 1:1, 1:3, 1:10, or 1:30. Controls without B cells (T only) were also measured. The cell counts were measured at different time points to monitor T cell expansion.

(C) Real-time cytotoxicity analysis of CFE-TCR-T cells against J82-NY targets with or without B cells. The RTCA (real-time cytotoxicity assay) method was used to analyze the cytotoxicity levels of CFE-TCR-T cells against J82-NY target cells in the presence or absence of B cells. J82-NY cells were seeded at a density of 1×10^5 (1E5) cells per well, and CAR-TCR-T cells were added at various concentrations ranging from 1×10^2 (1E2) to 1×10^5 (1E5) cells per well with or without B cells (1×10^5 [1E5]/well). The signals were recorded by the RTCA equipment and normalized.

(D) *In vivo* suppression of J82-NY tumor growth by CFE-TCR-T cells in the presence of CD19-positive cells. The antitumor effect of CFE-TCR-T cells in the presence of CD19-positive cells was evaluated *in vivo*. NSG mice were subcutaneously inoculated with J82-NY-GFP tumor cells. When the average tumor volume reached 100 mm_3, mice were randomly divided into four groups and infused intravenously with freezing medium (circle), MOCK- T (square), CFE-TCR-T (triangle), or CFE-TCR-T cells together with K562-CD19/CD86 (K562-CD19) cells (inverted triangle). Tumor volume was measured at different time points, and the results were presented as mean tumor volume (±SEM) for five mice per group. The *p* value less than 0.01 is represented by ** using the two-way analysis of variance with correction for multiple testing by the Bonferroni method.

(legend continued on next page)

other cytokines for 7–10 days. The modification rate and biological activity of transduced T cells were analyzed. On Figure 1B, it shows that transduced T cells expressing the ER can be recognized by PD-L1, and T cells expressing CEF can bind the CD19 protein. When such T cells are co-cultured with Raji overexpressing PD-L1, it is found that T cells armed with ER are more cytotoxic against PD-L1-positive tumor cells (Figure 1C). This effect is also seen for TCR-T cells. Conventional TCR-T cells, when stimulated by the corresponding major histocompatibility complex, are activated and released IL-2 (Figure 1D). However, this activation is significantly inhibited in the presence of tumor PD-L1 molecule. In contrast, TCR-T cells armed with ER, instead of being inhibited by PD-L1, are hyper-activated and released greater IL-2, demonstrating the intended reversal of the original immunosuppressive effect to produce an immunostimulatory signal through the action of the ER. Notably, ER contains only co-stimulatory molecules but does not contain the first signal CD3ζ, which forms an "AND gate" logic switch with the TCR containing CD3ζ and can only function when the TCR is stimulated by specific antigens in ER-armed T cells. ER in combination with tumor-specific T cells will enhance the killing efficacy of these tumor-specific T cells against PD-L1-expressing cancer cells, but not against PD-L1-expressing normal cells.

Our pilot study *in vitro* demonstrated that the ER-armed T cells can convert negative signals into stimulatory ones at the cellular level, promoting the release of more cytokines, thus restoring the function of previously suppressed tumor-specific T cells and resulting in greater tumor suppression. Based on these preliminary results, we used a xenotransplant tumor model established in immunodeficient mice to verify whether T cells armed with ER had stronger tumor-suppressive ability *in vivo*. Two animal models were used to evaluate the function of ER-modified CAR-T and TCR-T *in vivo*. Raji-PD-L1 is a human-derived lymphoma cell overexpressing PD-L1, which was implanted subcutaneously into immunodeficient mice (Figure 1E). When the tumor volume reached 100 mm^3, T cells were infused intravenously. Mice were then monitored for the tumor growth. In the PD-1+ CAR-T group, the tumor growth rate of mice was slower than that of the PD-1+ Mock-T group, but the tumor volume still showed a growth trend. In the PD-1+ ER-CAR-T group, the tumor growth of mice was significantly inhibited, and the tumor volume remained at a low level until day 25. This indicates that PD-1 + ER-CAR-T has a better inhibitory effect on Raji tumor growth than PD-1+ Mock-T and PD-1+ CAR-T. In a separate *in vivo* mouse tumor model established by injection of J82-ESO1, a PD-L1-positive bladder cancer line transfected with NY-ESO1 antigen, specific TCR-Ts against human leukocyte antigen HLA-A2-restricted NY-ESO1 with or without ER were infused for suppression of tumor growth. As seen in Figure 1F, specific TCR-T cells targeting to A2/ESO1 complex suppressed the growth of bladder cancer cells and J82-ESO1 in immunodeficient mice, and this suppression is significantly enhanced in the presence of ER. Furthermore, pharmacokinetic studies in mice showed that the number of ER-TCR-Ts in tumor tissue was significantly higher than that of the TCR-T group in tumor tissue, indicating that ER enhanced the infiltration of T cells into tumor tissues (Figure 1G).

It remains a challenge to expand tumor-specific T cells to a sufficient number for tumor therapies without the exhaustion of T cells. Successful CAR-T therapies against B lymphoma have demonstrated that pre-expansion CAR-Ts targeting CD19 can proliferate rapidly in the body. In order to facilitate the expansion of T cells in the B cell environment, we engineered an anti-CD19 CAR structure (CFE) on our T cells, and validation experiments were conducted on a TCR-T model with a pair of T cell receptors specifically recognizing HLA-A2/ESO1 complex on tumor cells (Figure 2A). We first evaluated the expansion ability of CFE-armed T cells (CFE-TCR-T) upon stimulation by co-cultured B cells. As shown in Figure 2B, the expansion of modified T cells in the presence of B cells correlates with the effector-to-target cell ratio, which is the best at 1:10 in this set of experiments. T cell expansion in the presence of B cells is better than that without B cells. In addition to increased cell number, we also evaluated whether the stimulation of B cell affected the function of modified T cells. In experiments with a real-time cytotoxicity assay (RTCA) using equipment with a monitorable real-time killing function (Figure 2C). 10^2 of TCR-T cells in the presence of B cells are comparable to 10^5 of T cells alone in terms of inhibition of the growth of J82-ESO1 target cells, demonstrating CFE as an effective driver for the expansion of TCR-Ts. In experiments with *in vitro* assays, the CFE is able to expand modified T cells with the help of B cells, reducing the number of T cells required to achieve the same degree of tumor cytotoxicity. We further tested T cells modified with CFE expanded *in vivo* by the stimulation of CD19-positive cells, to suppress tumor growth *in vivo*. As shown in Figure 2D, 1×10^6 of TCR-CFE-modified T cells, in the presence of CD19-positive K562 cells, significantly inhibit the growth of the target tumor cells J82-ESO1 *in vivo*, while neither CFE-TCR-modified T cells alone in the absence of $CD19^-$positive K562 cells nor CFE-modified unrelated T cells inhibit tumor growth effectively.

Having separately demonstrated that both ER and CFE improve tumor-specific T cell function, we further examined the combined effect of our PD-1+ T cell selection procedure with two strategies. The effect of therapeutic T cells with the 3-in-1 strategy (ScTILs)was evaluated for the treatment of personalized tumors (Figure 2E). First, we established a patient-derived xenograft (PDX) model to implant subcutaneously tumor cells dissected from an adenocarcinoma patient into immunodeficient NOD/scid/IL2r$\gamma^{-/-}$ (NSG) mice. The PD-1^+ T cells from the peripheral blood of the same patient modified with CFE and/or ER, and CD19-K562-stimulating cells, were co-infused into PDX mice with freezing medium or unmodified PD-1^+/PD-1^- T cells as controls. As seen in Figure 2E, PD-1-negative T cells

(E) ScTILs suppress self-origin tumor growth in a colon cancer PDX model. The antitumor effect of ScTILs was evaluated in PDX models established in NSG mice with tumor tissues obtained from patients with colon cancer. When the average tumor volume of PDX mice reached 100 mm^3, mice were randomly divided into 6 groups and infused with different T cells, and tumor volumes were measured at different time points. The results are presented as mean tumor volume (±SEM) of five mice for each group. The *p* value less than 0.01 is represented by ** using the two-way analysis of variance with correction for multiple testing by the Bonferroni method.

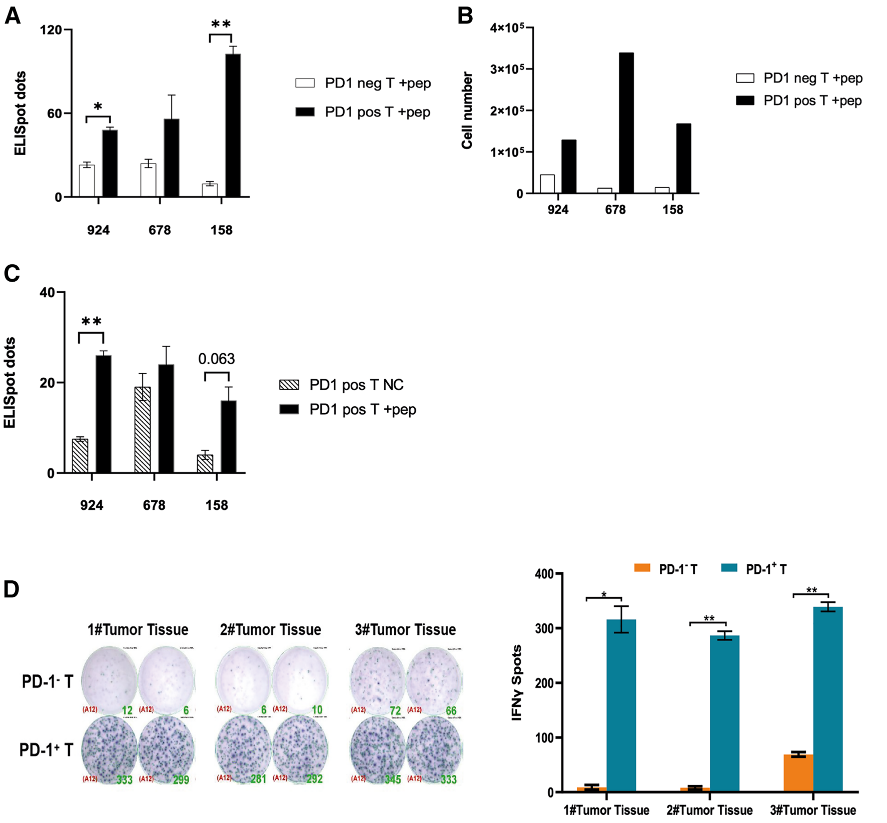

Figure 3. PD-1-positive T cells from peripheral blood contain neoantigen-specific T clones

(A) PD-1-positive T cells produce more IFN-γ-positive cells upon stimulation of neoantigen peptides. The p values less than 0.05 and less than 0.01 are represented by * and **, respectively.

(B) PD-1-positive T cells include the majority of IFN-γ captured T cells upon stimulation of peptides.

(C) PD-1-positive T cells generated more INF-γ spots on peptides stimulation than PD-1-negative T cells. The p value less than 0.01 is represented by **.

(D) Specific stimulation of PD-1-positive cells by self-tumor cells in colon cancer PDX models. The specific stimulation was investigated in PDX models constructed in NSG mice with tumor tissues obtained from patients with colon cancer. Tumor tissues were obtained from three PDX mice (designated 1#, 2#, and 3#) when the PDX model was passaged to the third generation. Peripheral blood PD-1-positive T cells and PD-1-negative T cells from the same patient were co-cultured with 1# to 3# tumor tissues, and IFN-γ-positive spots were detected by ELISPOT. The p values less than 0.05 and less than 0.01 are represented by * and **, respectively.

(filled square) without modification, similar to freezing medium, (filled black circle) do not suppress tumor growth. Unmodified PD-1 + T cells (filled triangle) also fail to suppress tumors, indicating that this class of cells lose the ability to inhibit tumors despite having the ability to specifically recognize heterogeneous tumors. PD-1^{+} cells transduced with ER (open circle and filled inverted triangle) show a recovery of T cell effect for the tumor suppression. T cells modified with CFE alone (filled diamond) are able to rapidly expand and overcome the PD-L1-PD-1 inhibitory effect through the co-stimulatory signal provided by the CD19 CAR structure, producing a stronger tumor suppression effect than PD-1+T cells modified by the ER alone. Finally, PD-1+ T cells modified with both ER and CFE (ScTILs, filled orange circle) produce the greatest effect of tumor suppression.

PD-1-positive cells from peripheral blood contain neoantigen-specific T cells

It has been demonstrated that PD-1 can be used as a surrogate marker to "enrich" neoantigen-specific T cells in the peripheral blood obtained from melanoma patients. To demonstrate that it is also true for biliary tract cancer (BTC), we have conducted experiments to isolate neoantigens and antigen-specific TCRs, similar to the study that has been done in melanoma. Since the cholangiocarcinoma patients participating in this study were not suitable for surgical treatment, it was impossible to obtain tumor tissues. We analyzed the presence of circulating tumor DNA in the whole blood of the patients and used them as candidate neoantigens. Circular tumor DNA (ctDNA) candidates derived from BTC donors (Table S1) as neoantigens were selected according to the procedure shown in STAR Methods. and screened peptide epitopes (Table S2) were synthesized according to procedures given in STAR Methods. PD-1+T and PD-1-T cells were isolated using an anti-PD-1 antibody from the peripheral blood mononuclear cells (PBMCs) of BTC patients. Both PD-1+T and PD-1-T cells were then incubated with an equal quantity of autologous PBMCs supplemented with the corresponding neoantigen peptide mixture at a concentration of 1 μg/mL. On day 6, the neoantigen peptide mixture was added once again. On day 7, interferon (IFN)-γ-secreting T cells were sorted using an IFN-γ-secreting T cells isolation kit (Miltenyi, Shanghai). An equal number of cells from different donors were tested for IFN-γ secretion by enzyme-linked immunospot (ELISpot) assays on day 8.

The results (Figure 3) reveal that PD-1-positive cells release more IFN-γ compared to PD-1-negative T cells after co-incubation with autologous antigen-presenting cells (APCs). This finding indicates that PD-1-positive cells represent an active T cell population (Figure 3A). We also isolated IFN-releasing cells from the stimulated T cells of donors using a commercial kit and found that the PD-1+ cell groups when incubated with peptide-loaded APCs yield the greatest number of cells (Figure 3B). Repeated analysis reveals that the PD-1-positive cell population, when stimulated by peptide-loaded APCs, produces the greatest number of IFN-γ-secreting T cells in comparison to PD-1-positive cells incubated with unloaded APCs (Figure 3C). In contrast, PD-1-negative cells produced scarcely any IFN-γ cells when incubated with peptide-loaded APCs (Figure 3A).

Based on the results of the finished experiments, the following can be concluded. (1) Neoantigen-specific clones do exist in

Table 1. Clinical and treatment characteristics of study subjects

Screen no.	Sex	Age	Diagnose	Clinical stage	Prior treatments	Leukapheresis	Dose
Information for qualified patients							
S001[a]	F	65	EHCC	IV, T3NxM1	radial surgery	N	N/A
S002[b]	M	52	ICC	IV, T3NxM1	TACE	Y	1×10^8
S003	F	57	ICC	IV, T3N1M1	PTCD; bile duct stenting	Y	1×10^8
S004	M	59	GBC	IV, T4N1M1	GEMOX chemotherapy	Y	1×10^8
S006	M	46	ICC	IIIB, T2N1M0	RFA plus surgery; TACE; PEI; RT 6000cGy/30F	Y	1×10^8
S009[b]	F	67	EHCC	IV	unknown	Y	1×10^8
S010	M	70	CC	IV, T1N1M1	TACE; surgery	Y	1×10^8
S014	M	53	CC	IIIB, T4N1M0	surgery; PTCD; GEMOX chemotherapy	Y	5×10^8
S016	M	66	GBC	IV, T4NxM1	surgery	Y	5×10^8
S023	F	54	ICC	IV, T3N2M0	radical surgery	Y	5×10^8
S024	F	58	ICC	IIIB, T4N1M0	TACE	Y	1×10^9
S025	M	66	ICC	IV, T4NxM1	radical surgery	Y	1×10^9
S028[c]	M	68	ICC	IV, T2NxM1	pembrolizumab + lenvatinib	Y	1×10^9
S029	M	62	ICC	IV, T3N2M0	surgery; ERCP	Y	1×10^9

[a]Peripheral blood apheresis was not performed due to disease progression.
[b]Cell therapy was not performed due to disease progression and baseline failure to meet requirements of cell infusion.
[c]Cell therapy was not performed due to prohibited drug by the protocol after enrollment.

PD-1-positive cells. Proliferation can be stimulated by neoantigen-specific APCs, and the proliferating T cell clones are accompanied by IFN-γ secretion. (2) Relatively abundant T cell clones can be obtained in T cells stimulated by neoantigen-loaded APCs, and these clones have the specific activation characteristics related to the IFN-γ secretion.

To further confirm that PD-1-positive T cells contain tumor-reactive cells, we used ELISpot assay to test cytokine release when specific T cells are stimulated by self-tumor cells. As shown in Figure 3D, only PD-1-positive PBLs from the patient are specifically activated to strongly release IFN-γ when exposed to autologous tumor cells, whereas PD-1-negative T cells release very little IFN-γ. These results confirm that PD-1-positive T cells contain the majority of the tumor-reactive T cell population.

The patients with advanced BTC have been successfully treated with ScTILs

As shown earlier, we have demonstrated that ScTILs, circulating PD-1-positive T cells modified with both CFE and ER, can suppress tumor growth both at the cellular level and in animal studies. A clinical trial (ScTILs-v2-001- 2019) was designed (Figure S1) based on aforementioned results and carried out in advanced-stage BTC patients heavily treated before with written informed consent, which was approved by the ethic committee of Peking Union Medical College Hospital (PUMCH, HS-2130).

As shown in Table 1, among the 29 patients screened, a total of 14 participants were enrolled in the dose-escalation phase of this trial from April 2020 to March 2021. Among the 14 enrolled patients, 1 did not receive peripheral blood apheresis due to disease progression; 10 of the 13 subjects who completed apheresis received ScTILs infusion, and the remaining 3 were not able to complete cell transfusion due to rapid disease progression resulting in failure to meet protocol infusion criteria (2 cases) or the use of prohibited drug according to protocol after enrollment (1 case). The baseline for clinical characterization of 14 patients is shown in Table 1. All 10 patients who completed ScTILs cell transfusion during the dose-escalation phase had received at least 1 treatment for the primary tumor or for a complication arising from the primary tumor. 2 of the 10 patients had received systemic chemotherapy, and all the rest were refused to accept chemotherapy. The median time from peripheral blood collection to cell infusion (vein-to-vein time) was 18 days (ranging from 15 to 21 days), with the exception of 2 patients whose vein-to-vein time was 41 and 29 days due to COVID-19 infection (S003) or additional bone scans after baseline examination (S010), respectively.

ScTILs therapy shows satisfied safety profile

In this early stage of clinical study, the analysis of safety data from 10 subjects with primary BTC treated with different dose groups of ScTILs shows that the most common (≥20% incidence) treatment-emergent adverse events (Table S4) include fever, digestive system disorder, liver and renal disfunction, myelosuppression, etc. The most common treatment-related adverse events (TRAE, Table S5) included fever, anorexia, lymphocyte count decrease, and headache; among them, fever occurs in 60% (6/10 cases) of patients. Additionally, the

incidence of decreased lymphocyte count is related to the mechanism of action of ScTILs *in vivo*.

As exhibited in Table S6, the incidence of grade smaller/equal to 3 TRAEs was 30% in all dose groups, and no grade 4 of TRAEs was observed; no cytokine release syndrome (CRS) or immune-related neurological syndrome (ICAN) occurred. The grade 3 or higher adverse events related to ScTILs infusion we observed includes decreased lymphocyte count (20%) and elevated blood conjugated bilirubin (10%). No patients (0/10) required tocilizumab or steroid treatment after ScTIL infusion. No event related to viral infection reactivation was observed. There were no ScTIL infusion-related allergic reactions or occurrence of autoimmune diseases observed in any of the 10 patients.

ScTILs expanded by stimulation of B cells in BTC patients

Genetically modified ScTIL cells contain a CAR structure that recognizes CD19-expressing B cells *in vivo*. The component activates a downstream cascade of effects by the CD28 and CD3ζ co-stimulatory domains, leading to the proliferation of T cells and the release of inflammatory factors and chemokines. A series of events ultimately lead to the expansion of ScTIL cells accompanied by killing CD19-positive B cells. The results of the dynamic changes in ScTILs vector gene copy number and B cell levels in the 7 subjects show that all of the patients have different degrees of B% decline within 2 weeks after ScTILs cell infusion; the B cell levels gradually recover after the vector gene copy number of ScTILs decrease in peripheral blood (Figure 4A). The analysis of the different groups shows that, as the dose of ScTIL cells increase, the reduction of B cell number is also more significant (Figure 4B). Thus, the level of B cell decline depends on ScTILs dose. Interestingly, the low dose of ScTILs seems to expand more quickly than its higher-dose counterpart according to their gene copy analysis (Figure 4C).

Efficacy analysis of patients treated with ScTILs

The clinical trial was initiated in late 2019 as an open-label, single-dose escalation infusion to observe and evaluate the tolerability and pharmacokinetic profile, as well as the safety and efficacy of ScTILs in the treatment of primary biliary duct malignancies.

In the dose-escalation phase, 10 subjects (8 treatment-naive and 2 previously treated) diagnosed with locally advanced or metastatic biliary duct malignancies were enrolled to receive cellular infusion in three different dose groups: 1×10^8 (4 cases), 5×10^8 (3 cases), and 1×10^9 (3 cases) seen in Table 1. The clinical response was evaluated at 6 and 12 weeks after infusion with computed tomography (CT)/MRI scanning. The indicators for the efficacy used were OS (overall survival) and PFS (progression-free survival) as of August 2022, as well as surrogate indicators overall response rate and duration of response evaluated with imaging evaluation at 6 and 12 weeks after ScTIL infusion by investigators according to the criteria of Response Evaluation Criteria in Solid Tumors version 1.1 (RECIST v.1.1). For efficacy evaluation, a total of 9 subjects (3 in each of the low-, medium-, and high-dose groups) underwent imaging evaluation at 6 weeks, and 4 subjects (1 in the low-dose group and 3 in the medium-dose group) went on to complete 12-week imaging evaluation (Table 2). The best efficacy among patients acquired imaging evaluation was SD (Figures 5A–5C and S3).

Efficacy is further analyzed post hoc across different dosage groups. Comparing the low-dose group (1×10^8), the medium-dose group (5×10^8), and the high-dose group (1×10^9), the following observations were made: PFS is 3.39, 6.83, and 1.35 months, respectively; OS is 14.62, 18.3, and 3.15 months, respectively (Table S7A); DCR at 6 weeks is 50% (2/4), 100% (3/3), and 0% (0/3), respectively; and the DCR at 12 weeks is 25% (1/4), 67% (2/3), and 0% (0/3), respectively (Table S7B; Figure S2A). The efficacy of the high-dose group (1×10^9) is significantly inferior to that of the low-dose and medium-dose groups, showing basically no curative effect at all. To explore why the high-dose group (1×10^9) did not show significant efficacy, we have conducted experiments to compare the expression levels of markers of T cell exhaustion such as PD-1, TIM-3, and LAG-3 among T cells in different dose groups, as shown in Figure S4. However, there are not any meaningful differences among them. The memory phenotypes of T cells from different dosage groups were also analyzed, and results are shown in Figure S5.

Clinical outcomes (OS, PFS, and DCR) in 10 subjects across three dose groups (1.0×10^8, 5.0×10^8, and 1.0×10^9) are summarized in supplemental tables and figures. Table S7A and Figure S2A show median OS (3.2–18.3 months) and PFS (1.35–6.83 months) with no significant dose differences ($p = 0.107$ for OS and $p = 0.085$ for PFS), while Table S7B and Figure S2B show that the 5.0×10^8 dose had the highest DCR at 6 weeks (100%) and 12 weeks (66.7%), versus 0% for 1.0×10^9; total cohort DCR was 50% (6 weeks) and 30% (12 weeks). Censoring/missing data arose from loss to follow-up, subsequent treatment, early death, or study withdrawal. Despite no significant OS/PFS differences, 5.0×10^8 trended toward better DCR, though small sample sizes and missing data limit interpretation. Considering the high-dose group (1×10^9) as the placebo control and combining the low-dose and medium-dose groups (1×10^8–5×10^8) for the 1×10^8–5×10^8 dose group ($n = 7$) and the control 1×10^9 dose group ($n = 3$), PFS is 5.42 months versus 1.35 months. OS is 18.3 months versus 3.15 months (Table S7C; Figure S2D). DCR at 6 weeks (Table S7D) is 71% (5/7) versus 0% (0/3). DCR at 12 weeks is 43% (3/7) versus 0% (0/3). The efficacy analysis among groups with baseline B% reveals that a significant inverse correlation is observed between dosage and efficacy when the dosage exceeds the threshold.

As shown in Tables S8A and S8B and Figure S2C, for the group with normal B% ($n = 3$) versus the group with relatively low B% ($n = 7$): PFS is 6.83 months versus 1.4 months, OS is 18.3 months versus 4.0 months, DCR at 6 weeks is 100% (3/3) versus 29% (2/7), and DCR at 12 weeks is 100% (3/3) versus 0% (0/7). However, in this study, there were only three patients with normal B%. To avoid the conclusion being biased due to the small sample size, we moderately lower the criterion for relatively low B% to 50% of the lower limit of the normal value, so as to expand the sample group with normal B% to seven people and observe whether the conclusion remains consistent. For the adjusted group with normal B%—the group of patients with B% higher than 50% of the lower limit of the normal value (labeled as B%N50, $n = 7$) versus the adjusted control group—the group of patients with B% lower than 50% of the lower limit

CellPress
OPEN ACCESS

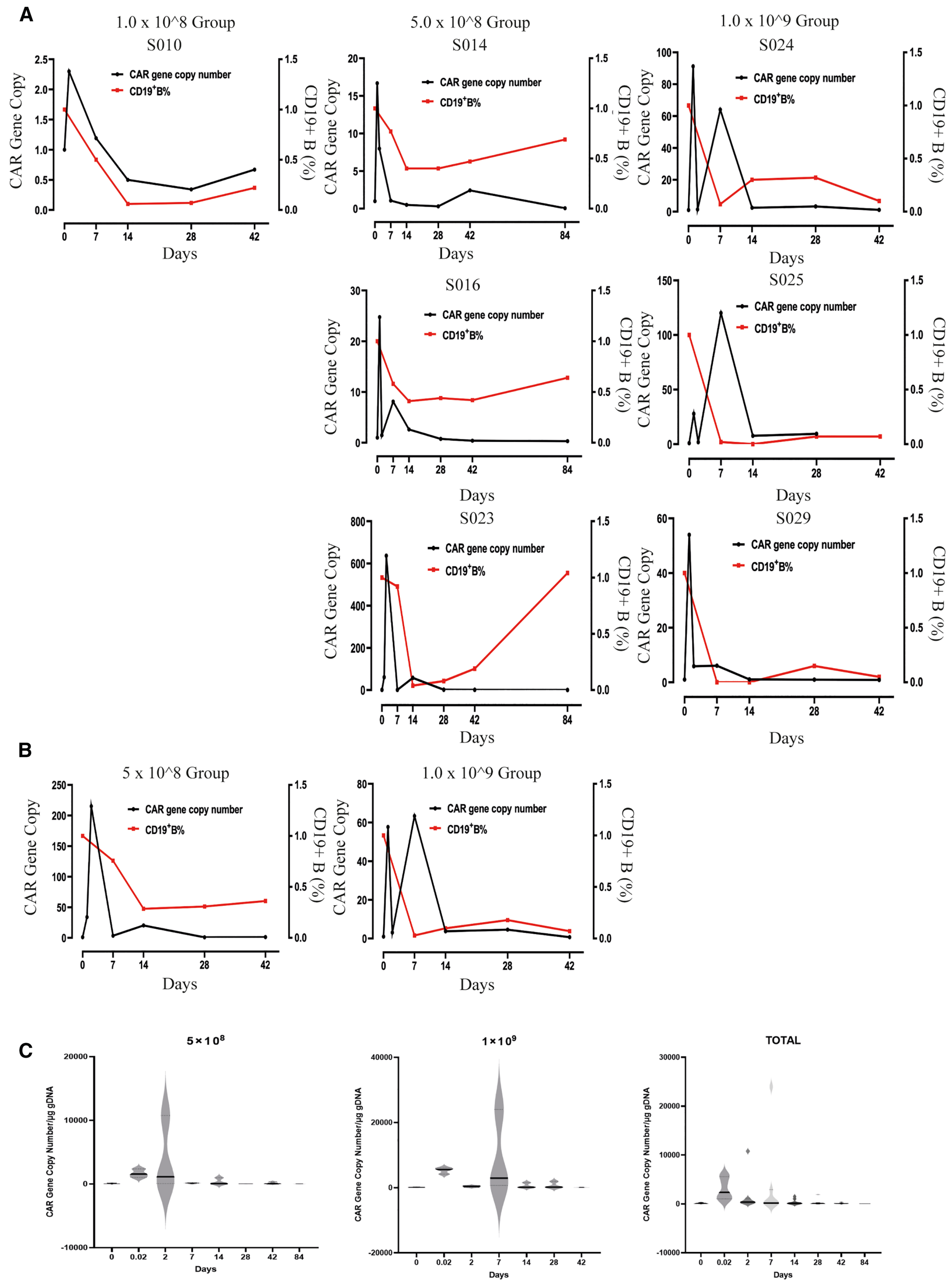

Figure 4. The relative changes of CD19 CAR copy number and CD19$^+$B% to baseline at different time points after infusion
(A and B) CD19 CAR copy number and CD19$^+$B% curve in each patient (A) and groups summary (B) with dynamic monitoring.
(C) The average CD19 CAR copy number in each group and all patients.

Table 2. Clinical and treatment characteristics of study subjects

Dosage	Subject	Groups	Dosage + B%	B cell (%)	6W	12W	PFS (Mth)	Med PFS	OS (Mth)	Med OS	12W DCR
Efficacy comparison between patients with normal and low B cells											
1×10^8	S003	test	normal	12.9	SD	SD	5.4	7.0	14.5	18.3	80%
5×10^8	S016	test	normal	11.1	SD	SD	12.0	7.0	18.3	18.3	80%
5×10^8	S014	test	normal	11.0	SD	SD	7.0	7.0	19.8	18.3	80%
5×10^8	S023	test	normal	7.5	SD	PD	2.7	7.0	8.4	18.3	80%
1×10^8	S010	test	normal	5.4	SD	SD	24.2	7.0	24.2	18.3	80%
1×10^8	S006	control	low	3.5	PD	PD	1.4	1.4	1.4	3.2	0%
1×10^8	S004	control	low	2.3	N/A	N/A	0.2	1.4	0.2	3.2	0%
1×10^9	S024	control	normal	8.6	PD	PD	1.2	1.4	4.0	3.2	0%
1×10^9	S025	control	normal	6	PD	PD	1.3	1.4	2.6	3.2	0%
1×10^9	S029	control	low	2	PD	PD	1.5	1.4	3.2	3.2	0%

PFS (Mth), progression-free survival (months); Med PFS, median progression-free survival; OS (Mth), overall survival (months); Med OS, median overall survival; 12w DCR, 12 weeks of disease control rate.

of the normal value (labeled as B%L50, $n = 3$), PFS is 5.42 months versus 1.4 months, OS is 18.3 months versus 3.2 months, DCR at 6 weeks is 71% (5/7) versus 0% (0/3), and DCR at 12 weeks is 43% (3/7) versus 0% (0/3). (Tables S8C and S8D; Figure S2D).

We have further analyzed treatment efficacy by combining both dosage and B%. Since ScTILs hardly had any curative effect on patients with a high dose (1×10^9) and those with B% below 50% of the lower limit of the normal value, we might as well regard patients with a high dose (1×10^9) and those with B% below 50% of the lower limit of the normal value as the control group ($n = 5$) and consider other patients, namely those with a dose of 1×10^8–5×10^8 and B% higher than 50% of the lower limit of the normal value ($n = 5$), as the sample group. Then, the comparison results (Figure 5D) are as follows: the sample group (labeled as B%N50 & 1×10^8–5×10^8, $n = 5$) versus the control group (labeled as B%L50 & 1×10^9, $n = 5$): PFS is 6.83 months versus 1.4 months, OS is 18.3 months versus 3.2 months, DCR at 6 weeks is 100% (5/5) versus 0% (0/5), and DCR at 12 weeks is 60% (3/5) versus 0% (0/5).

The pharmacokinetics of B cell-to-lymphocyte ratios

Due to the introduction of CAR construct targeting CD19 in the 3-in-1 strategy, it was predicted that, same as CAR-T for B cell-related malignancy, B cells would be significantly reduced after ScTILs infusion and the reduction in B cells would continue for a considerable period of time as ScTILs *in vivo* expansion proceed. The proportion of B cell to lymphocytes (B%) was monitored in patients in this study. As can be seen from the data presented in Figure 4A and Table S10, the baseline proportion of B cells to leukocytes (B%) of 70% (7/10) patients was below 9.0%, the lower limit of normal (LLN). The level of B% on average was minimized in 89% and 11% patients by day 14 and day 7, respectively, after ScTILs infusion; besides, the B% level dropped to zero in three patients by day14 but were gradually recovered in the following several weeks. All patients (100% 4/4) who completed the 12-week assessment experienced a transient decline in B% levels and returned to normal levels at 12 weeks (Figure 6A). The results indicate that the elimination of normal B cell induced by ScTILs treatment is transient and reversible.

We obtained the data of the baseline and dynamic changes post-ScTILs infusion of immunoglobulins (Ig) including IgA, IgG, and IgM, which indirectly reflect the function of B lymphocytes, from 9 patients (Figure 6A). During the 12-week observation, 22%, 11%, and 11% of the subjects showed a decrease in IgA, IgG, and IgM levels but were still >50% of baseline values, respectively. No statistically or clinically significant abnormal immunoglobulin results were observed in these patients; besides, none of the reductions met the diagnostic criteria for hypoglobulinemia. The combined results of dynamic monitoring of B cells and immunoglobulins following ScTIL transfusion suggest that B cell reduction that occurred following ScTIL transfusion in the low- or medium-dose group is transient and does not result in severe hypoglobulinemia.

Cytokines

In this clinical trial, the pharmacodynamic response to ScTILs infusion was also assessed by measuring changes in serum concentrations of cytokines or chemokines for 6 to 12 weeks post-ScTIL infusion. Detailed information of cytokine changes is shown in Figure 6B. The baseline levels and dynamic monitoring data of cytokines were obtained in 7 patients. Elevation of cytokines occurred mainly within 7 days post-infusion, with greater than 3-fold elevation occurring in IL-6 (28%) and IL-10 (14%), while no greater than 10-fold cytokine elevations were observed, none of which met the diagnostic criteria for CRS. The cytokine level of these patients returned to baseline at the follow-up period of 14 days post-infusion.

DISCUSSION

As mentioned earlier, there are three major barriers to effective T cell therapy for solid tumors: cancer cell heterogeneity, hostile TME, and limited T cell expansion. To break these barriers, this study utilized a 3-in-1 strategy involving cTIL, ER, and CFE to produce ScTIL cells for the treatment of biliary malignancies known to be aggressive and lethal. ScTILs have been demonstrated to significantly prolong PFS and OS in patients with advanced biliary tumors while current medicine struggles to

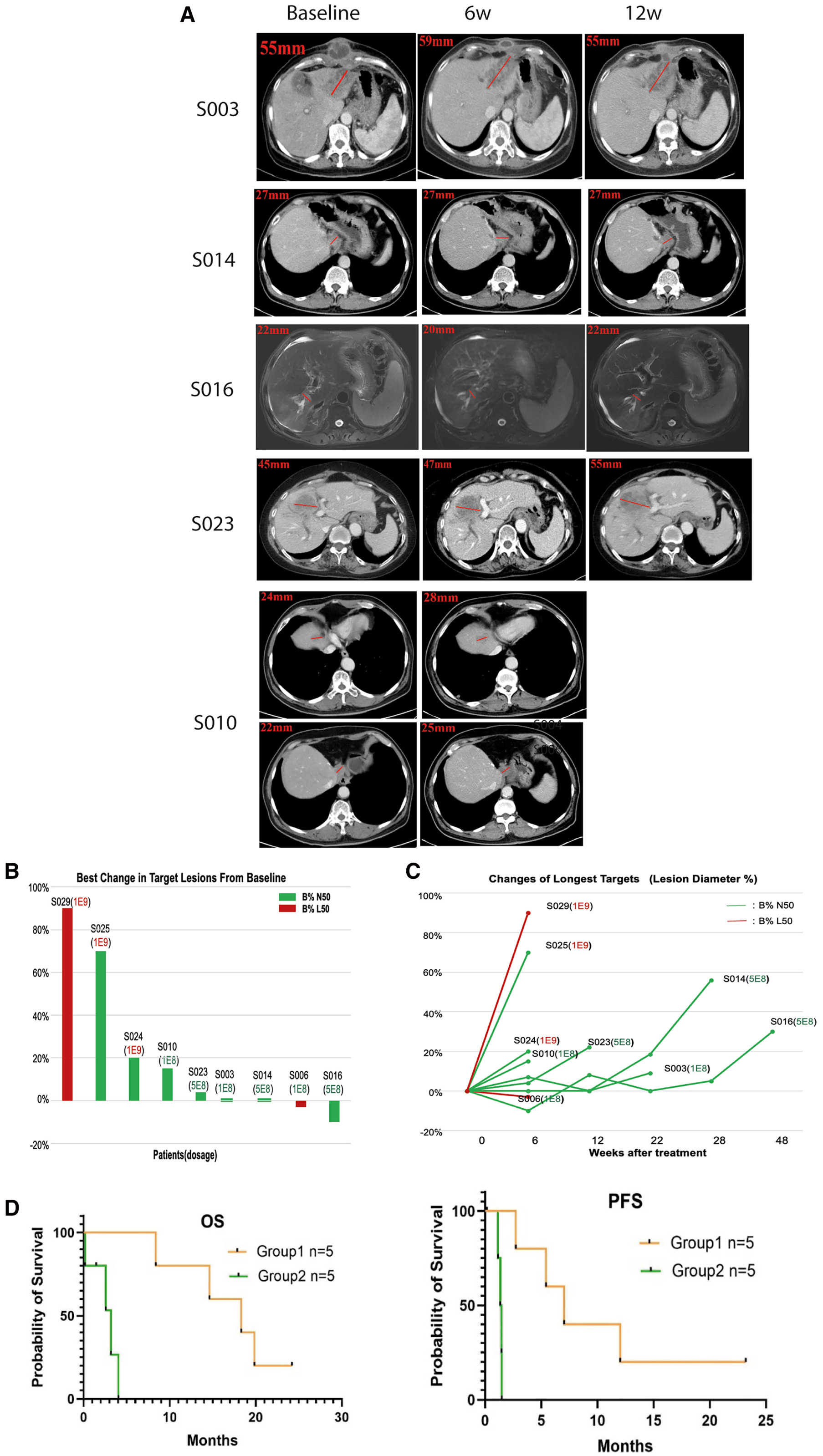

(legend on next page)

provide long-term PFS and OS for advanced patients who do not respond to the last-line therapy.

In this study, ScTILs therapy shows several safety advantages over other immunotherapies like TILs, CAR-T/TCR-T, and PD-1 monoclonal antibodies. Compared with TILs, ScTILs infusion does not need pre-treatment chemotherapy to deplete lymphocytes nor high concentration of IL-2, thus avoiding related toxicities. Differing from conventional CAR-T and TCR-T, ScTILs introduce CFE to target B cells for *in vivo* expansion without long-time culture *in vitro*. Versus PD-1 monoclonal antibodies, ScTILs have an ER that can theoretically activate T cells more strongly; the "AND gate" design of ER prevents common adverse reactions seen with PD-1 monoclonal antibodies. Due to these advantages, no treatment-related grade 4+ adverse events occurred, and neither CRS nor ICANS was observed. Plus, the effect on B cells was limited and temporary.

Efficacy comparison across groups shows significant differences. The medium-low-dose groups ($1–5 \times 10^8$) outperformed the high-dose group (1×10^9), likely due to cell exhaustion from excessive *in vitro* expansion in the high-dose group or the high T/B ratio affecting CFE *in vivo* expansion. Efficacy is also directly related to baseline B% level; patients with baseline B% > 50% LLN had an OS of 18.3 months, five times longer than those with baseline B% < 50% LLN (3.2 months), consistent with the CFE mechanism. Thus, ScTILs' optimal dose is $1–5\ 1 \times 10^8$ and is suitable for patients with baseline B% > 50% LLN. Of the current first-line standard treatments for biliary tract tumors, "gemcitabine plus cisplatin" has an OS of 11.7 months, and updated treatments combining anti-PD-1/PD-L1 monoclonal antibodies with chemotherapy only achieve OS of 12.7 and 12.8 months, respectively.

Regarding tumor neoantigen heterogeneity, endogenous T cells are polyclonal and specific to various tumor epitopes. TILs, as endogenous T cells, can be effectively stimulated by tumor tissues and suppress heterogeneous tumors. Thus, identifying endogenous neoantigen-specific T cells with surface markers is valuable. The current literature indicates that PD-1 expression may identify peripheral T cells recognizing tumor neoantigens. In this study, PD-1+T cells (cTIL) from BTC patients' peripheral blood were used to address tumor neoantigen heterogeneity and as ScTILs precursor cells. *In vitro* experiments, like co-culturing tumor lysates and antigen peptides with cTILs and ELISpot detection, showed that cTILs had higher specificity for heterogeneous tumors and neoantigens than PD-1-negative cells. But using cTILs as an alternative to TILs in BTC patients needs further validation. The ER in ScTILs can reverse immune checkpoint inhibitory signals and activate cTILs for enhanced anti-cancer function (proven *in vitro* and in animal experiments).

We chose *in vivo* autologous T cell expansion via endogenous B cell co-stimulation instead of *in vitro* expansion. This cuts costs, shortens preparation time, and prevents ScTILs senescence from excessive *in vitro* growth. The experimental treatment uses a CFE construct targeting CD19 for *in vivo* T cell co-stimulation and rapid proliferation, at the expense of limited, transient B cell depletion. ScTILs co-localize with B cells, causing temporary B cell death, but immunoglobulin levels remained unaffected during treatment, and B cell counts returned to normal at 3 months. ScTILs rely on *in vivo* B cells for T cell expansion, eliminating the need for pre-transfusion lymphodepletion. In this study, patients with normal baseline B cells did not require pre-treatment chemotherapy, achieved good efficacy, avoided chemotherapy-related toxicity, and had improved quality of life. Unlike TIL therapy, ScTILs do not need ultra-high-dose IL-2 after infusion, sparing patients from its severe side effects.

ScTILs therapy has successfully tackled the three major obstacles stated earlier to effective immunotherapy for solid tumors. However, this study is only a preliminary exploration of ScTILs. Out of the patients, 80% had not received prior first-line therapy as they were disinclined to undergo due to chemotherapy (only 2 out of 10 patients had received front-line systemic therapy). Moreover, the assessment of effectiveness was based on a *post hoc* analysis of a small, biased subgroup of patients. Additionally, among the 14 initially enrolled patients, 4 did not receive treatment (3 due to rapid disease progression and 1 due to prohibited drug use). Their exclusion from the efficacy analysis may have biased the reported OS.

Limitations of the study

While this study offers valuable insights into ScTILs therapy for cancer, several limitations persist. First, the specific types and quantities of tumor neoantigen-reactive T cells involved in ScTILs remain unclear. Second, the small sample size limits conclusions about efficacy in biliary tract and other solid tumors; more data are needed on infused T cell survival and distribution. Additionally, while ScTILs improved OS in some patients, they did not induce widespread tumor shrinkage, highlighting a need to enhance this response. Finally, despite a shorter production cycle than TILs, rapid progression of advanced cancers still led to some patient dropouts, necessitating further cycle optimization.

Figure 5. Efficacy analysis of ScTILs treatment

(A) Target lesions' CT/MRI imaging of baseline and different evaluation time points in stable disease patients. For S003, S014, S016, and S023, sample liver lesions at the beginning, week 6, and week 12 of the treatment were shown. Both of the liver lesion and lymph nodes of S010 at the beginning of treatment and week 6 were shown. The red lines refer to the long diameter of target liver lesions or short diameter of target lymph nodes. Efficacy was assessed by calculating the sum of the diameters of all target lesions and comparing this aggregate value with the corresponding baseline measurements.

(B) Changes of longest targets (lesion diameter %) in treated patients. In the 9 patients who could be evaluated for efficacy, 5 had stable disease (SD) and 4 had progressive disease (PD). Among them, S006 was assessed as having progressive disease (PD) due to the appearance of new lesions. B% N50 (in blue): baseline B cell proportion $\geq$ 50% LLN; B% L50 (in red): baseline B cell proportion < 50% LLN.

(C) Best change of target lesions from baseline. (1) S006 was evaluated as PD due to the appearance of new lesions at week 6; (2) S010 withdrew from the trial after week 6, without reaching the progression free survival (PFS); (3) S003 was evaluated as PD due to the appearance of new lesions at week 22. B% N50: baseline B cell proportion $\geq$ 50% LLN; B% L50: baseline B cell proportion < 50%LLN.

(D) Kaplan-Meier analysis of overall survival (OS) and progression-free survival (PFS) for comparison between patients with sufficient B cells (test group) and patients with insufficient B cells including low B cells and high dosage of ScTILs (control group).

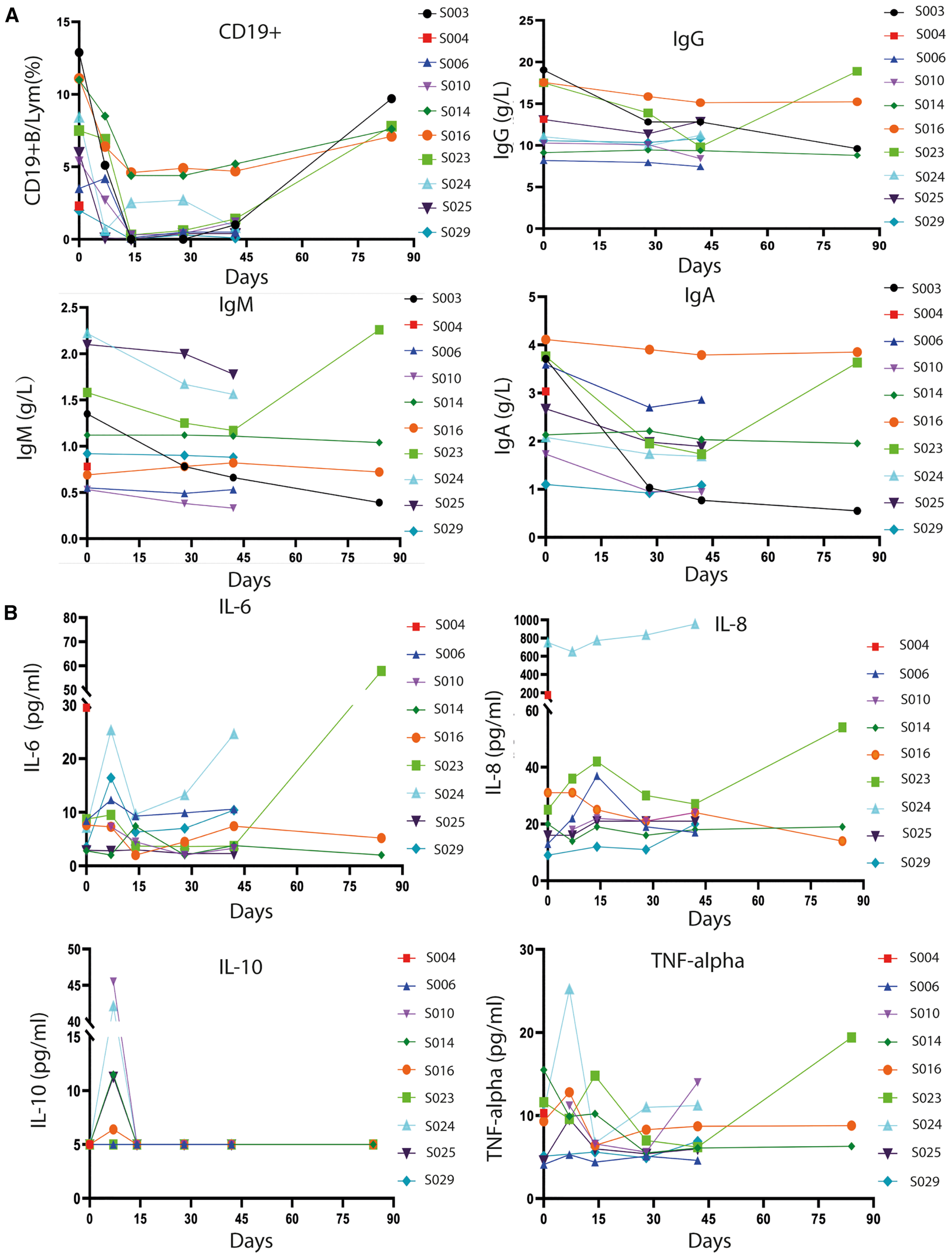

Figure 6. Pharmacodynamic index of B cells and concentrations of immunoglobulins and cytokines in treated patients with ScTILs

(A) Percentage change of CD19-positive B cells in total lymphocytes and the concentration changes of each immunoglobulin subclass in treated patients during observed time.

(B) The concentrations (pg/mL) of serum cytokines of IL-6, IL-8, IL-10, and TNF-α in patients before and after ScTILs infusion for different time intervals.

RESOURCE AVAILABILITY

Lead contact

Further information and requests for resources and reagents should be directed to and will be fulfilled by the lead contact, Bin Gao (bgao2004@icloud.com).

Materials availability

Reagents generated in this study will be made available on request. However, a payment and/or a completed materials transfer agreement may be required if there is potential for commercial application.

Data and code availability

- All experimental data generated in this study will be available upon request from the lead contact.
- This study does not generate original code.
- Any additional information required to re-analyze the data reported in this paper is available from the lead contact upon request.

ACKNOWLEDGMENTS

We thank Dr. Sean Gao at Cambridge for his critical reading of the manuscript and Dr. Yasong Wu and his team at the Discovery Research, Chineo Medical Technology Co., Ltd. for their contributions to the results presented in Figure 3. This research is partly supported by the Beijing Science and Technology Commission under grant number Z221100007922039 to B.G. and Beijing Natural Science Foundation (L248022), National High Level Hospital Clinical Research Funding (2022-PUMCH-B-128 and 2025-PUMCH-D-001), CAMS Innovation Fund for Medical Sciences (CIFMS) (2022-I2M-C&T-A-003), CAMS Innovation Fund for Medical Sciences (CIFMS) (2021-I2M-1-061 and 2021-I2M-1-003), CSCO-Hengrui Cancer Research Fund (Y-HR2019-0239, Y-HR2020MS-0415, and Y-HR2020QN-0414), CSCO-MSD Cancer Research Fund (Y-MSDZD2021-0213), and National Ten-thousand Talent Program to H.Z.

AUTHOR CONTRIBUTIONS

W.G., B.G., and H.Z. conceptualized the project. X.W., J.Z., X.Y., X.M., and B.L. were responsible for the methodology. X.W., J.Z., X.Y., X.M., and B.L. carried out the investigation. B.G., W.G., and H.Z. supervised the project. All authors gave feedback to the manuscript drafted by B.G. The manuscript draft was further developed and reviewed and edited by B.G. and W.G.

DECLARATION OF INTERESTS

J.Z., X.M., B.L., B.G., and W.G. are employees of Chineo Medical Technology Co., Ltd. B.G. and W.G. serve on the company's management board, and W.G. is a shareholder of Chineo Medical Technology Co., Ltd. J.Z., B.G., and W.G. are inventors on patent applications that cover portions of the work described in this article.

STAR★METHODS

Detailed methods are provided in the online version of this paper and include the following:

- KEY RESOURCES TABLE
- EXPERIMENTAL MODEL AND STUDY PARTICIPANT DETAILS
  - *In vivo* xenograft experiments
  - Generation of colon adenocarcinoma PDX
  - Study participants
- METHOD DETAILS
  - Cell culture conditions
  - Construction of the enhance receptor (ER), CAR for expansion (CFE) and ER-T2A-CFE
  - Lentiviral vector production
  - Peripheral blood mononuclear cells and tumor tissues
  - Isolation of human peripheral blood mononuclear cells and B cells
  - Isolation of PD-1 positive T lymphocytes from PBMCs of cancer patients
  - Transduction and expansion of PD-1$^+$ and PD-1$^-$ T lymphocytes
  - Colon adenocarcinoma PDX cells
  - Monitoring of cell growth using real-time unlabelled cell analyser (RTCA)
  - *In vitro* co-culture experiments
  - ELISPOT assay
  - Measurement of ScTILs and CD19$^+$ B cells *in vivo*
  - Measurement of cytokines in serum by multiplex analysis
- QUANTIFICATION AND STATISTICAL ANALYSIS
- ADDITIONAL RESOURCES

SUPPLEMENTAL INFORMATION

Supplemental information can be found online at https://doi.org/10.1016/j.xcrm.2025.102349.

Received: February 12, 2024
Revised: January 21, 2025
Accepted: August 13, 2025
Published: September 18, 2025

REFERENCES

1. Umut, Ö., Gottschlich, A., Endres, S., and Kobold, S. (2021). CAR T cell therapy in solid tumours: a short review. Memo *14*, 143–149. https://doi.org/10.1007/s12254-021-00703-7.
2. Tan, H., Bao, J., and Zhou, X. (2015). Genome-wide mutational spectra analysis reveals significant cancer-specific heterogeneity. Sci. Rep. *5*, 12566. https://doi.org/10.1038/srep12566.
3. Bruin, E.C., Taylor, T.B., and Swanton, C. (2013). Intra-tumour heterogeneity: lessons from microbial evolution and clinical implications. Genome Med. *5*, 101. https://doi.org/10.1186/gm505.
4. Dagogo-Jack, I., and Shaw, A.T. (2018). Tumour heterogeneity and resistance to cancer therapies. Nat. Rev. Clin. Oncol. *15*, 81–94. https://doi.org/10.1038/nrclinonc.2017.166.
5. Rosenberg, S.A., Spiess, P., and Lafreniere, R. (1986). A new approach to the adoptive immunotherapy of cancer with tumour-infiltrating lymphocytes. Science *233*, 1318–1321. https://doi.org/10.1126/science.3489291.
6. Monberg, T.J., Borch, T.H., Svane, I.M., and Donia, M. (2023). TIL Therapy: Facts and Hopes. Clin. Cancer Res. *29*, 3275–3283. https://doi.org/10.1158/1078-0432.CCR-22-2428.
7. Kim, T.Y., von Eschenbach, A.C., Filaccio, M.D., Hayakawa, K., Parkinson, D.R., Balch, C.M., and Itoh, K. (1990). Clonal analysis of lymphocytes from tumour, peripheral blood, and nontumourous kidney in primary renal cell carcinoma. Cancer Res. *50*, 5263–5268.
8. Ahmadzadeh, M., Johnson, L.A., Heemskerk, B., Wunderlich, J.R., Dudley, M.E., White, D.E., and Rosenberg, S.A. (2009). Tumour antigen-specific CD8 T cells infiltrating the tumour express high levels of PD-1 and are functionally impaired. Blood *114*, 1537–1544. https://doi.org/10.1182/blood-2008-12-195792.
9. Gros, A., Robbins, P.F., Yao, X., Li, Y.F., Turcotte, S., Tran, E., Wunderlich, J.R., Mixon, A., Farid, S., Dudley, M.E., et al. (2014). PD-1 identifies the patient-specific CD8(+) tumour-reactive repertoire infiltrating human tumours. J. Clin. Investig. *124*, 2246–2259. https://doi.org/10.1172/JCI73639.
10. Gros, A., Tran, E., Parkhurst, M.R., Ilyas, S., Pasetto, A., Groh, E.M., Robbins, P.F., Yossef, R., Garcia-Garijo, A., Fajardo, C.A., et al. (2019). Recognition of human gastrointestinal cancer neoantigens by circulating PD-1+ lymphocytes. J. Clin. Investig. *129*, 4992–5004. https://doi.org/10.1172/JCI127967.

CellPress
OPEN ACCESS

11. Liu, Z., Li, J.P., Chen, M., Wu, M., Shi, Y., Li, W., Teijaro, J.R., and Wu, P. (2020). Detecting Tumour Antigen-Specific T Cells via Interaction-Dependent Fucosyl- Biotinylation. Cell *183*, 1117–1133. https://doi.org/10.1016/j.cell.2020.09.048.

12. Li, T., Zhao, L., Yang, Y., Wang, Y., Zhang, Y., Guo, J., Chen, G., Qin, P., Xu, B., Ma, B., et al. (2021). T Cells Expanded from PD-1(+) Peripheral Blood Lymphocytes Share More Clones with Paired Tumour-Infiltrating Lymphocytes. Cancer Res. *81*, 2184–2194. https://doi.org/10.1158/0008-5472.CAN-20-2300.

13. John, L.B., Devaud, C., Duong, C.P.M., Yong, C.S., Beavis, P.A., Haynes, N.M., Chow, M.T., Smyth, M.J., Kershaw, M.H., and Darcy, P.K. (2013). Anti-PD-1 antibody therapy potently enhances the eradication of established tumours by gene-modified T cells. Clin. Cancer Res. *19*, 5636–5646. https://doi.org/10.1158/1078-0432.CCR-13-0458.

14. Chong, E.A., Melenhorst, J.J., Lacey, S.F., Ambrose, D.E., Gonzalez, V., Levine, B.L., June, C.H., and Schuster, S.J. (2017). PD-1 blockade modulates chimeric antigen receptor (CAR)-modified T cells: refueling the CAR. Blood *129*, 1039–1041. https://doi.org/10.1182/blood-2016-09-738245.

15. Rupp, L.J., Schumann, K., Roybal, K.T., Gate, R.E., Ye, C.J., Lim, W.A., and Marson, A. (2017). CRISPR/Cas9-mediated PD-1 disruption enhances anti-tumour efficacy of human chimeric antigen receptor T cells. Sci. Rep. *7*, 737. https://doi.org/10.1038/s41598-017-00462-8.

16. Cherkassky, L., Morello, A., Villena-Vargas, J., Feng, Y., Dimitrov, D.S., Jones, D.R., Sadelain, M., and Adusumilli, P.S. (2016). Human CAR T cells with cell-intrinsic PD-1 checkpoint blockade resist tumour-mediated inhibition. J. Clin. Investig. *126*, 3130–3144. https://doi.org/10.1172/JCI83092.

17. Gargett, T., Yu, W., Dotti, G., Yvon, E.S., Christo, S.N., Hayball, J.D., Lewis, I.D., Brenner, M.K., and Brown, M.P. (2016). GD2-specific CAR T Cells Undergo Potent Activation and Deletion Following Antigen Encounter but can be Protected from Activation-induced Cell Death by PD-1 Blockade. Mol. Ther. *24*, 1135–1149. https://doi.org/10.1038/mt.2016.63.

18. Adusumilli, P.S., Zauderer, M.G., Rivière, I., Solomon, S.B., Rusch, V.W., O'Cearbhaill, R.E., Zhu, A., Cheema, W., Chintala, N.K., Halton, E., et al. (2021). A Phase I Trial of Regional Mesothelin-Targeted CAR T-cell Therapy in Patients with Malignant Pleural Disease, in Combination with the Anti-PD-1 Agent Pembrolizumab. Cancer Discov. *11*, 2748–2763. https://doi.org/10.1158/2159-8290.CD-21-0407.

19. Sang, W., Wang, X., Geng, H., Li, T., Li, D., Zhang, B., Zhou, Y., Song, X., Sun, C., Yan, D., et al. (2022). Anti-PD-1 Therapy Enhances the Efficacy of CD30-Directed Chimeric Antigen Receptor T Cell Therapy in Patients with Relapsed/Refractory CD30+ Lymphoma. Front. Immunol. *13*, 858021. https://doi.org/10.3389/fimmu.2022.858021.

20. Chong, E.A., Alanio, C., Svoboda, J., Nasta, S.D., Landsburg, D.J., Lacey, S.F., Ruella, M., Bhattacharyya, S., Wherry, E.J., and Schuster, S.J. (2022). Pembrolizumab for B- cell lymphomas relapsing after or refractory to CD19-directed CAR T-cell therapy. Blood *139*, 1026–1038. https://doi.org/10.1182/blood.2021012634.

21. Liu, X., Ranganathan, R., Jiang, S., Fang, C., Sun, J., Kim, S., Newick, K., Lo, A., June, C.H., Zhao, Y., and Moon, E.K. (2016). A Chimeric Switch-Receptor Targeting PD-1 Augments the Efficacy of Second-Generation CAR T Cells in Advanced Solid Tumours. Cancer Res. *76*, 1578–1590. https://doi.org/10.1158/0008-5472.CAN-15-2524.

22. Liu, H., Lei, W., Zhang, C., Yang, C., Wei, J., Guo, Q., Guo, X., Chen, Z., Lu, Y., Young, K.H., et al. (2021). CD19-specific CAR T Cells that Express a PD-1/CD28 Chimeric Switch-Receptor are Effective in Patients with PD-L1-positive B-Cell Lymphoma. Clin. Cancer Res. *27*, 473–484. https://doi.org/10.1158/1078-0432.CCR-20-1457.

23. Wickström, S., and Lövgren, T. (2019). Expansion of Tumour-Infiltrating Lymphocytes from Melanoma Tumours. Methods Mol. Biol. *1913*, 105–118. https://doi.org/10.1007/978-1-4939-8979-9_7.

24. Ghassemi, S., Nunez-Cruz, S., O'Connor, R.S., Fraietta, J.A., Patel, P.R., Scholler, J., Barrett, D.M., Lundh, S.M., Davis, M.M., Bedoya, F., et al. (2018). Reducing Ex Vivo Culture Improves the Antileukemic Activity of Chimeric Antigen Receptor (CAR) T Cells. Cancer Immunol. Res. *6*, 1100–1109. https://doi.org/10.1158/2326-6066.CIR-17-0405.

STAR★METHODS

KEY RESOURCES TABLE

REAGENT or RESOURCE	SOURCE	IDENTIFIER
Antibodies		
biotin anti-human PD-1 #329934; RRID: AB_2563527	Biolegend	www.biolegend.com
Anti-human CD3 #300306; RRID: AB_314042	Biolegend	www.biolegend.com
Anti-human PD-1 #329908; RRID: AB_940475	Biolegend	www.biolegend.com
OKT3 #T210;	Takara	www.takara.com
7-AAD #420404	Biolegend	www.biolegend.com
Bacterial and virus strains		
Lentivirus	This manuscript	N/A
Biological samples		
Tumor tissue and peripheral blood mononuclear cells (PBMCs)	This manuscript	N/A
Chemicals, peptides, and recombinant proteins		
human IL-2	Quanqi	www.quanqi.com
Cell Serum Replacement #A2596102	Gibco	www.gibco.com
Liberase	Roche	www.roche.com
Deposited data		
Patient data	This manuscript	N/A
Experimental models: Cell lines		
J82 #HTB-1	ATCC	www.atcc.com
K562 #CCL243	ATCC	www.atcc.com
Raji	National infrastructure of cell resources, China	www.cellresource.cn
Experimental models: Organisms/strains		
NOD/scid/IL2r$\gamma^{-/-}$ (NSG) mice	Beijing IDMO Co., Ltd	N/A
Oligonucleotides		
WPRE-F: 5′- GGCACTGACAATTCCGTGGT-3′	This manuscript	N/A
WPRE-R: 5′- AGGGACGTAGCAGAAGGACG-3′	This manuscript	N/A
WPRE-Probe: 5′-FAM- AGCCA TGGAAAGGACGTCAGCTTC-BHQ1-3′	This manuscript	N/A
ALB-F:5′- AGTGCACTTGT TGAGCTCGTG-3′	This manuscript	N/A
ALB-R:5′- GCAAAGCAG GTCTCCTTATCG-3′	This manuscript	N/A
ALB-Probe:5′-FAM- TGAAAGCT GTTATGGATGATTTCGCAGC-BHQ1-3′	This manuscript	N/A
Software and algorithms		
GraphPad Prism	GraphPad	www.graphpad.com
NetMHCpan-4.1	DTU Health Tech.	https://services.healthtech.dtu.dk/services/NetMHCpan-4.1

EXPERIMENTAL MODEL AND STUDY PARTICIPANT DETAILS

In vivo xenograft experiments

All experiments were approved by the Committee of Chineo Medical Technology Co. Ltd. on Animal and Technical Ethics. 6 to 8 weeks old of female NOD/scid/IL2r$\gamma^{-/-}$ (NSG) mice were purchased from Beijing IDMO Co., Ltd. and housed in SPF facility. Tumor cells (1×10^6, J82-NY-ESO-1-GFP, HLA: 0201) in a solution of PBS/Matrigel (1:1) were injected subcutaneously and bilaterally in the

flanks of mice (n = 5 per group). Tumor volumes were measured and calculated as: volume = 0.5 × length × width.[2] When J82-NY-ESO-1-GFP tumors reached an average volume of 100 mm^3, the mice were divided into different groups, and injected with different T cells intravenously into the tail.

Generation of colon adenocarcinoma PDX

Tumor pieces (15–60 mm3) from colon adenocarcinoma patient were obtained from surgery and subcutaneously implanted into 6-week-old female NOD/scid/IL2r$\gamma^{-/-}$ (NSG) mice (Beijing IDMO Co., Ltd.). Animals were housed in air-filtered flow cabinets with a 12:12 light/dark cycle, and food and water were provided *ad libitum*. Upon growth of the engrafted tumors, a tumor piece was implanted into a new recipient mouse for the model perpetuation. At the 4th passage, the tumors were sliced into 3 mm × 3 mm × 3mm fragments and inoculated subcutaneously on the right flank of 6-8-week-old female NSG mice. When the tumor burden was approximately 100 mm^3, the mice were randomly separated into three groups (n = 5) and injected intravenously (i.v.) with different T cells (1×10^7 T cells/mouse). The tumor dimensions were measured three times weekly with calipers, and the tumor volumes were calculated using the formula (volume = 0.5 × length × $width^2$), where the length is the greatest longitudinal diameter and the width is the greatest transverse diameter.

Study participants

Details of the demographics of the patients who received cell therapy are shown in Table S3, of which 70% and 30% were male and female respectively; the mean age was 59 years (range 46–70 years). The clinical diagnosis was bile duct cancer in 8 cases and gallbladder cancer in 2 cases; the number of cases in each dose group was as follows: 4 cases were in the low dose (1×10^8), 3 cases in the medium dose (5×10^8) and 3 cases in the high dose (1×10^9). Patients were recruited following the obtaining of written informed consent, and the study protocol—including the consent procedure—was approved by the Ethics Committee of Peking Union Medical College Hospital (PUMCH; Approval No.: HS-2130).

METHOD DETAILS

Cell culture conditions

Various cell lines were used in this study, including J82, an HLA-A2+ urinary bladder carcinoma cell line (HTB-1, ATCC). NY-ESO-1 or PD-L1 was lentivirally transduced into J82 to produce J82-NY-ESO-1 (J82-NY) or J82-NY-PDL1, respectively. K562, a chronic myelogenous leukemia cell line (CCL243, ATCC) in which a CD19 and CD86 expression cassette was incorporated by lentiviral transduction to produce K562-CD19/86. Additionally, Raji, a human B lymphoblastoid cell line originally derived from a patient with Burkitt lymphoma (National infrastructure of cell resources, China), was lentivirally transduced with PD-L1 to produce Raji-PDL1. Lastly, the Click beetle green (CBG) gene was incorporated into Raji-PDL1 to produce Raji-PDL1-CBG by lentiviral transduction. All cell lines were cultured in DMEM (Gibco) or RPMI 1640 (Gibco) supplemented with 10% heat-inactivated FBS, 100 U/mL penicillin, 100 μg/mL streptomycin sulfate, and 1% L-glutamine.

Construction of the enhance receptor (ER), CAR for expansion (CFE) and ER-T2A-CFE

The enhance receptor (ER) was constructed by fusing a scFv against PD-L1 (Atezolizumab) with the intracellular domain of the CD28 molecule (GenBank: NM_006139.4; AA141-220, transmembrane and cytoplasmic domains). We used an anti-CD19 scFv to create 2nd generation CD19 CARs harboring 4-1BB costimulatory domains as CAR for expansion (CFE). ER or CFE was subcloned into the viral vectors upstream of a T2A sequence that was followed by NY-ESO-1 specific TCR for producing ER-TCR-T and CD19 CAR-TCR-T cell respectively. The enhance receptor was linked to CD19 CAR by the T2A sequence, and was sub-cloned into the lentiviral vector for producing super circulation TILs (ScTILs).

Lentiviral vector production

The 293T (ATCC) cells were seeded and cultured overnight. The cells were co-transfected with pDM2.G and pMDL-Gag, pRev, lentiviral vector using lipofectamine 2000 transfection. After 24 and 48 h, the medium containing the lentivirus was collected, filtered through a 0.45 μm filter, ultra-centrifuged, re-suspended in PBS, and stored at −80°C.

Peripheral blood mononuclear cells and tumor tissues

Tumor tissue and peripheral blood mononuclear cells (PBMCs) were collected from patients with informed written consent and following the Ethical Committee's guidelines of Peking Union Medical College Hospital (. The study protocol was approved by Peking Union Medical College Hospital.

Isolation of human peripheral blood mononuclear cells and B cells

Human peripheral blood mononuclear cells (PBMCs) were isolated using Ficoll-paque density-gradient centrifugation. B cells were enriched using immunomagnetic selection with human CD19 microbeads (Miltenyi Biotec) according to the manufacturer's instruction.

Isolation of PD-1 positive T lymphocytes from PBMCs of cancer patients

PBMCs from patients was first incubated with anti-PD-1 antibody (329934, Biolegend, 10μg/ml) for 1 h at 4°C to ensure all PD-1^+ cells bound with the anti-PD-1 antibody and then subsequently incubated with anti-Biotin Microbeads (20 μL for 10^7 PBMCs) for 15 min at 4°C before transferring into MS or LS column for cell separation. The magnetic separation procedure was repeated once to increase purity.

Transduction and expansion of PD-1^+ and PD-1^- T lymphocytes

The sorted PD-1^+ and PD-1^- T lymphocytes were cultured in X-VIVO15 serum-free medium supplemented with 2.5% Immune Cell Serum Replacement (A2596102, Gibco) and IL-2 (3000 IU). The cells were stimulated with anti-CD3/anti-CD28 at a 1:3 cell to bead ratio for overnight and then were transduced with lentiviral vectors at an MOI of approximately 2. Cells were counted and fed with full X-VIVO15 media every 2 days and once T cells appeared to become quiescent, as determined by both decreased growth kinetics and cell size, they were used either for functional assays or cryopreserved.

Colon adenocarcinoma PDX cells

Tissue from colon adenocarcinoma PDXs was dissociated and digested for 45 min at 37°C with Liberase (0.15 mg/mL, Roche), passed through a 70 μm cell strainer (BD Biosciences) and cultured in DMEM, supplemented with 10% FBS,100 U/mL penicillin, 100 mg/mL streptomycin sulfate, and 1% L-glutamine at 37°C in 5% CO_2. PDX cells were routinely grown to confluence and dissociated using Accutase.

Monitoring of cell growth using real-time unlabelled cell analyser (RTCA)

Cell growth was continuously monitored for 48 h using the xCELLigence RTCA MP instrument (Roche). 1×10^5 J82-NY cells were seeded per well into the E-plates 16, and initial attachment and growth were continuously monitored for approximately 24 h. Different amount of CD19 CAR-TCR-T cells with or without 1×10^5 B cells were then added to the wells and responses were continuously recorded for approximately additional 24 h. Each amount in each CD19 CAR-TCR-T cells was repeated in duplicate in order to increase the reliability of biological experiment data. The CI reading frequency was once per 15 min until completion of experiment.

In vitro co-culture experiments

J82-NY cells and T cells were co-cultured at a 1:1 cell ratio. For cytokine production assays, supernatants were collected 24 h after co-culture and were assessed for human IL-2 and IFN-γ using the DuoSet ELISA Development Kit (R&D Systems). For killing assays, T cells were co-cultured with Raji-PDL1-CBG cells at a different cell ratio for 16 h. Following co-culture, wells were washed, and remaining tumor cells were lysed with 1X cell lysis buffer for 30 min. The luciferase activity in the lysates was analyzed using the Luciferase Assay System on a GloMax Multi Detection System (Promega). Results are reported as percent killing based on luciferase activity in wells with tumor, but no T cells. (% killing = 100-((RLU from well with effector and target cell coculture)/(RLU from well with target cells) x100)). Effector-to-target ratios represent total T cells per target cell.

ELISPOT assay

T cells were thawed into T cell medium supplemented with 3000 IU IL-2 two days before co-culture with PDX cells. Before each co-culture, PDX cells and T cells were washed and replaced with cytokine-free T cell medium. Typically, equal volumes (100 μL) of T cells and PDX cells were mixed together in a 96-well plate. 2×10^4 T cells were co-cultured with 1×10^5 PDX cells. All co-cultures were performed in the absence of exogenously added cytokines. Plate-bound OKT3 (1 μg/mL; Miltenyi Biotec) was used as a positive control. Media were used as negative controls. The second laboratory (Villa Marelli Institute) used the commercial T-SPOT. TB assay in accordance with the procedures recommended by the manufacturer. The individual spots were counted using an automated image analysis system, ELISPOT reader (AID-GmbH, Strassberg, Germany).

Measurement of ScTILs and CD19$^+$ B cells *in vivo*

After ScTILs infusion, the presences of ScTIL and CD19$^+$ B cells in blood were measured by RT-qPCR or flow cytometry. Blood samples were obtained from patients before and at intervals after ScTIL cell infusion and flow cytometry was performed to identify CD19$^+$ cells. PB samples collected from patients were cleared of erythrocytes using an ammonium-chloride-potassium buffer and were immediately stained with anti-human CD19 antibody (555415, BD) to detect CD19$^+$ B cells on PBL. Data were acquired with a CytoFLEX flow cytometer (Beckman) and analyzed with FlowJo software (FlowJo).

Q-PCR was used to analyze the dynamic changes of ScTILs in the blood. Genomic DNA was isolated directly from whole-blood using magnetic bead-based blood genomic DNA extraction kit (Tiangen, China) according to the manufacturer's instructions and stored at −80°C before use. The primer/probe combination specific for WPRE gene sequences was as follows:WPRE-F: 5′-GG CACTGACAATTCCGTGGT-3'; WPRE-R: 5′-AGGGACGTAGCAGAAGGACG-3'; WPRE-Probe: 5′-FAM-AGCCATGGAAAGGACGT CAGCTTC-BHQ1-3'; The primer/probe combination specific for non-transcribed genomic sequence upstream of the albumin (ALB) gene sequences was as follows: ALB-F:5′-AGTGCACTTGTTGAGCTCGTG-3'; ALB-R:5′-GCAAAGCAGGTCTCCTTATCG-3'; ALB-Probe:5′-FAM-TGAAAGCTGTTATGGATGATTTCGCAGC-BHQ1-3'. Q-PCR analysis on genomic DNA samples was performed in bulk using 6–300 ng of genomic DNA/time-point, 7500 Real-Time PCR System (Thermo Fisher) and a validated assay to detect the

integrated WPRE transgene sequence that was an element on the ER-CFE lentivirus vector. To determine the copy number per unit DNA, a 7-point standard curve was generated consisting of 6 to 6×10^6 copies plasmid standards. The number of copies of plasmid present in the standard curve was verified using Q-PCR system with the same WPRE primer/probe set. Each data-point (sample, standard curve) was evaluated in duplicate. To control for the quality of interrogated DNA, a parallel amplification reaction was performed using a primer/probe combination specific for a non-transcribed genomic sequence upstream of the ALB gene as described. These amplification reactions generated a correction factor to adjust for calculated versus actual DNA input. Copies of transgene per microgram DNA were calculated according to the formula: Copies/microgram genomic DNA = copies calculated from WPRE standard curve x correction factor/amount DNA evaluated (μg).

Measurement of cytokines in serum by multiplex analysis

Serial serum samples were obtained at different intervals before and after administration of ScTILs. Analysis of serum cytokines, including IL-6, IL-8, IL-10 and TNFα were carried out using the BD Cytometric Bead Array (CBA) Human Th1/Th2 Cytokine Kit II.

QUANTIFICATION AND STATISTICAL ANALYSIS

For statistics, GraphPad Prism software was used. All variables reported are continuous. Differences between experimental conditions were analyzed using the unpaired two-sided Student’s t test. For comparison of experimental conditions of individual mice, the Mann-Whitney U test was used. p values under 0.05 were considered statistically significant (*$p < 0.05$, **$p < 0.01$, ***$p < 0.001$). For *in vivo* experiments, differences between groups were analyzed using two-way analysis of variance with correction for multiple testing by the Bonferroni method. Overall survival was analyzed by log rank test. Survival was defined as days from tumor induction until natural death or until tumor size became greater than 5 times that of the initial size. Data are shown as mean values ±SD of a minimum of three biological replicates or independent experiments, as indicated. All statistical tests were two-sided.

ADDITIONAL RESOURCES

The study is registered with the Chinese Clinical Trial Registry (ChiCTR) under the registration number ChiCTR2000029738, and the registration information can be accessed at http://www.chictr.org.cn/.

Cell Reports Medicine, Volume *6*

Supplemental information

Therapeutic T cells with 3-in-1 strategy for the treatment of biliary tract cancer

Xueshuai Wan, Jie Zhao, Xiaobo Yang, Xianing Mou, Bing Liu, Bin Gao, Weiyue Gu, and Haitao Zhao

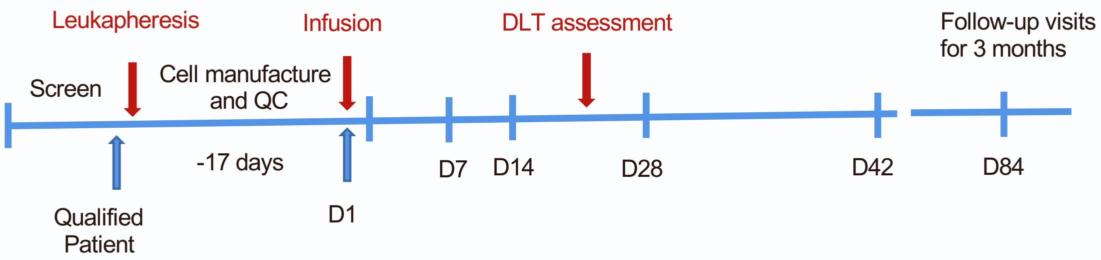

Supplemental Figure S1. Schematic Illustration of the Clinical Study Design. Related to Figure 4,5,6

The figure illustrates the timeline of the clinical trial, encompassing key stages such as screening for qualified patients, leukapheresis, cell manufacturing and quality control (QC), infusion, dose-limiting toxicity (DLT) assessment, and follow-up visits spanning 3 months. The timeline is marked with specific time points: Day 1 (D1) for infusion, and subsequent follow-up assessments on Day 7 (D7), Day 14 (D14), Day 28 (D28), Day 42 (D42), and Day 84 (D84). Leukapheresis is scheduled 17 days prior to infusion.

Supplemental Table S1 HLA typing of Donors. Related to Figure 3A

Donor	HLA typing					
924	HLA-A*02:07	HLA-A*24:02	HLA-B*15:01	HLA-B*46:01	HLA-C*01:02	HLA-C*04:01
678	HLA-A*11:01	HLA-A*30:01	HLA-B*07:02	HLA-B*13:02	HLA-C*06:02	HLA-C*07:02
158	HLA-A*02:07	HLA-A*31:01	HLA-B*40:02	HLA-B*46:01	HLA-C*01:02	HLA-C*04:01

Supplemental Table S2 Candidate neoantigen peptides. Related to Figure 3A

Donor	Gene	Amino acid change	Mutation type	Peptide name	Peptide Seq.	HLA	%Rank_EL(Affinity) #
924	IER5	p.L15V	Missense	924-1-IER5	VSISVGKIY	HLA-B*46:01	0.124
	PLSCR2	p.L238F	Missense	924-2-PLSCR2	SFDEQIVV	HLA-C*04:01	0.036
				924-3-PLSCR2	GVDFEITSF	HLA-C*04:01	0.449
158	XIRP2	p.L1869P	Missense	158-1-XIRP2	NMPATLKSL	HLA-A*02:07	0.431
	TAOK3	p.Q722Tfs*14	Frameshift	158-2-TAOK3	MEMQIKKTV	HLA-B*40:02	0.059
	MADD	p.Y1285C	Missense	158-3-MADD	RVYLCEGLLGR	HLA-A*31:01	0.11
	ATP6V0A4	p.S611R	Missense	158-4-ATP6V0A4	HAPRILIHF	HLA-B*46:01	0.288
	OXER1	p.I275S	Missense	158-5-OXER1	IVSSGLTIRNR	HLA-A*31:01	0.345
	MGA	p.A2372V	Missense	158-6-MGA-1	TTAVHTQSF	HLA-B*46:01	0.107
				158-7-MGA-2	VAHLKTTAV	HLA-C*01:02	0.443
	MGA	p.H2373Qfs*32	Frameshift	158-8-MGAfs-1	AAQTHSHSNSR	HLA-A*31:01	0.469
				158-9-MGAfs-2	VERLHQFL	HLA-B*40:02	0.475
				158-10-MGAfs-3	NSRPVLTSL	HLA-B*46:01	0.281
678	SACS	p.L3395V	Missense	678-1-SACS	YFNCNVNHL	HLA-C*07:02	0.364
	DCSTAMP	p.R439W	Missense	678-2-DCSTAMP	RWLSLYLTK	HLA-A*30:01	0.266
	LOC729159	p.P99Pfs*22	Frameshift	678-3-LOC-1	VVWSPPRGR	HLA-A*11:01	0.312
				678-4-LOC-2	WSPPRGRNL	HLA-C*06:02	0.363
	SRRM2	p.Q1828Qfs*36	Frameshift	678-5-SRRM2-1	RQFPDLLSTPK	HLA-A*11:01	0.276
				678-6-SRRM2-2	HSRSPARQFPDLL	HLA-B*07:02	0.076
				678-7-SRRM2-3	TPNQSEAFSLTHI	HLA-B*07:02	0.256
	CCT8L2	p.C72Vfs*30	Frameshift	678-8-CCT8L2-1	SSMAPPGSR	HLA-A*30:01	0.39
				678-9-CCT8L2-2	HPQGPGAGAPSSM	HLA-B*07:02	0.146
	CUBN	p.F832Ffs*68	Frameshift	678-10-CUBN-1	RLVAVPFWVLLK	HLA-A*11:01	0.043
				678-11-CUBN-2	KIKSIAVQTYL	HLA-A*30:01	0.374
				678-12-CUBN-3	KAKSFSSTSLSLK	HLA-A*30:01	0.45
				678-13-CUBN-4	LPTVKQIMLRLV	HLA-B*07:02	0.211
				678-14-CUBN-5	IRSPFFLTCIL	HLA-B*07:02	0.413
				678-15-CUBN-6	STSPKAKSF	HLA-C*06:02	0.308
	NEURL	p.D29Dfs*107	Frameshift	678-16-NEURL1-1	APRGHPQNL	HLA-B*07:02	0.002
				678-17-NEURL1-2	SPMPPQAEAL	HLA-B*07:02	0.009
				678-18-NEURL1-3	APSHAAAL	HLA-B*07:02	0.079
				678-19-NEURL1-4	YRGPLPRHFSPM	HLA-B*07:02	0.117
				678-20-NEURL1-5	GPHLRASQA	HLA-B*07:02	0.483
				678-21-NEURL1-6	LQQPPGPHL	HLA-B*13:02	0.078
				678-22-NEURL1-7	GQLLQRHHL	HLA-B*13:02	0.138
				678-23-NEURL1-8	SQAEDHQEAV	HLA-B*13:02	0.338

*, The mutated amino acids are marked in bold, italic and underlined.
#, Rank threshold for strong binding peptides: <0.500.
From NetMHCpan-4.1,https://services.healthtech.dtu.dk/services/NetMHCpan-4.1/

Supplemental Table S3. Demographics of patients who received ScTIL cells therapy. Related to Figure 5 and Table 1

Characteristic	Statistic	Low (N=4) 1.0×10^8	Medium (N=3) 5.0×10^8	High (N=3) 1.0×10^9	Total (N=10)
Age	N	4	3	3	10
	Mean(SD)	58(9.83)	57.67(7.23)	62(4)	59.1(7.17)
	Median	59	54	62	58.5
	Min, Max	46, 70	53, 66	58, 66	46,70
	Q1~Q3	54.25, 61.75	53.5, 60	60, 64	54.75, 65
Sex	All	4	3	3	10
	Male (%)	3(75.0)	2(66.7)	2(66.7)	7(70.0)
	Female (%)	1(25.0)	1(33.3)	1(33.3)	3(30.0)
Nationality	All	4	3	3	10
	Han Nationality (%)	3(75.0）	3(100)	3(100)	9(90.0)
	Others (%)	1(25.0)	0	0	1(10.0)
Baseline Weight (Kg)	N(missing)	3(1)	3(0)	3(0)	9(1)
	Mean(SD)	76.5(16.26)	67.5(7.50)	53.5(24.06)	66.09(13.25)
	Median	78.8	67.5	57.5	65
	Min, Max	65,88	60,75	43,60	43,88
	Q1~Q3	70.75, 82.25	63.75, 71.25	50.3, 58.8	60, 75

Supplemental Table S4 Treatment emergent adverse events, TEAE. Related to Figure 5

AE term	Frequency	Incidence (N=10)	Rate
Metabolic and Nutritional Diseases	14	6	60
Hypoalbuminemia	2	2	20
Hyponatremia	1	1	10
Loss of appetite	2	2	20
Hyperuricemia	4	2	20
Hypertriglyceridemia	1	1	10
Hyperglycemia	2	2	20
Hypernatremia	1	1	10
Hyperkalemia	1	1	10
Systemic diseases and various reactions at the administration site	9	6	60
Weak	2	1	10
Fever	7	6	60
Various tests	46	6	60
Elevated C-reactive protein	1	1	10
Elevated alanine aminotransferase	3	1	10
High neutrophil count	1	1	10
Low neutrophil count	4	2	20
Weight loss	1	1	10
Prolonged prothrombin time	1	1	10
Elevated aspartate aminotransferase	2	2	20
Increased anti-transglutaminase antibodies	1	1	10
Prolonged activated partial thromboplastin time	1	1	10
Low lymphocyte count	3	3	30
High white blood cell count	1	1	10
Low white blood cell count	3	1	10
Elevated fibrin D-dimer	1	1	10
Elevated conjugated bilirubin	5	3	30
Low platelet count	4	2	20
Elevated alkaline phosphatase	4	3	30
Elevated creatinine	3	2	20
Elevated bilirubin	7	4	40
Various nervous system diseases	3	3	30
Dizziness	1	1	10
Headache	2	2	20
Respiratory, thoracic and mediastinal diseases	2	1	10
Respiratory failure	1	1	10
Hypoxia	1	1	10
Infections and infestations	2	2	20
Upper respiratory infection	1	1	10
Abdominal wall abscess	1	1	10
Skin and Subcutaneous Tissue diseases	1	1	10
Rash	1	1	10
Mental illness	1	1	10
Insomnia	1	1	10
Gastrointestinal Diseases	6	5	50
lower gastrointestinal bleeding	1	1	10
constipate	1	1	10
nausea	1	1	10
stomach ache	1	1	10
bloating	1	1	10
noninfectious gingivitis	1	1	10
Blood and Lymphatic System Diseases	3	3	30
Anemia	3	3	30

Supplemental Table S5 Treatment related adverse events, TRAE. Related to Figure 5

AE term	Frequency	Incidence (out of 10)	Rate
Metabolic and Nutritional Diseases	2	2	20
Loss of appetite	2	2	20
Systemic diseases and various reactions at the administration site	7	6	60
Fever	7	6	60
Blood tests	13	5	50
Elevated alanine aminotransferase	3	1	10
Decreased neutrophil count	1	1	10
Decreased lymphocyte count	2	2	20
Elevated conjugated bilirubin	2	1	10
Low platelet count	1	1	10
Elevated alkaline phosphatase	2	2	20
Elevated bilirubin	2	1	10
Nervous system diseases	2	2	20
Headache	2	2	20
Mental Illness	1	1	10
Insomnia	1	1	10
Gastrointestinal Diseases	3	3	30
Constipation	1	1	10
Nausea	1	1	10
Bloating	1	1	10

Supplemental Table S6 Treatment related adverse events (TRAE) by grade. Related to Figure 5

Adverse event	Incidence N (%) *				Frequency M (%) **			
(N=10)	Cumulative Cases	Grade 1	Grade 2	>= Grade 3	Cumulative frequency	Grade 1	Grade 2	>=Grade 3
All	8(80.0)	2(20.0)	3(30.0)	3(30.0)	28(100.0)	15(53.6)	8(28.6)	5(17.9)
Fever	6(60.0)	4(40.0)	2(20.0)	0(0.0)	7(25.0)	5(17.9)	2(7.1)	0(0.0)
Loss of appetite	2(20.0)	2(20.0)	0(0.0)	0(0.0)	2(7.1)	2(7.1)	0(0.0)	0(0.0)
Headache	2(20.0)	2(20.0)	0(0.0)	0(0.0)	2(7.1)	2(7.1)	0(0.0)	0(0.0)
Decreased Lymphocyte count	2(20.0)	0(0.0)	0(0.0)	2(20.0)	2(7.1)	0(0.0)	0(0.0)	2(7.1)
Elevated bilirubin	1(10.0)	0(0.0)	1(10.0)	0(0.0)	2(7.1)	1(3.6)	1(3.6)	0(0.0)
Elevated alkaline phosphatase	1(10.0)	0(0.0)	0(0.0)	1(10.0)	2(7.1)	0(0.0)	1(3.6)	1(3.6)
Low platelet count	1(10.0)	0(0.0)	1(10.0)	0(0.0)	1(3.6)	0(0.0)	1(3.6)	0(0.0)
constipate	1(10.0)	1(10.0)	0(0.0)	0(0.0)	1(3.6)	1(3.6)	0(0.0)	0(0.0)
nausea	1(10.0)	1(10.0)	0(0.0)	0(0.0)	1(3.6)	1(3.6)	0(0.0)	0(0.0)
bloating	1(10.0)	0(0.0)	1(10.0)	0(0.0)	1(3.6)	0(0.0)	1(3.6)	0(0.0)
Elevated alanine aminotransferase	1(10.0)	0(0.0)	1(10.0)	0(0.0)	3(10.7)	2(7.1)	1(3.6)	0(0.0)
Decreased neutrophil count	1(10.0)	0(0.0)	1(10.0)	0(0.0)	1(3.6)	0(0.0)	1(3.6)	0(0.0)
Insomnia	1(10.0)	1(10.0)	0(0.0)	0(0.0)	1(3.6)	1(3.6)	0(0.0)	0(0.0)
elevated conjugated bilirubin	1(10.0)	0(0.0)	0(0.0)	1(10.0)	2(7.1)	0(0.0)	0(0.0)	2(7.1)

*The denominator is all subjects who have used the drug at least once, and each subject only selects the most severe level for calculation.

** The denominator is the frequency of all AEs, and the numerator is the frequency of each grade.

Supplemental Table S7. OS, PFS and DCR analysis for treated subjects in different dosage groups or in Low & Middle-dose, and High-dose groups. A. OS and PFS analysis for treated subjects in 1.0×10^8, 5.0×10^8, 1.0×10^9 groups. Related to Figure 5; B. DCR results of treated subjects in 1.0×10^8, 5.0×10^8, and 1.0×10^9 dose groups; C. OS and PFS analysis for treated subjects in Low& Middle-dose, and High-dose groups; D. DCR results of treated subjects in Low & Middle-dose, and High-dose groups. Related to Figure 5.

S7A OS and PFS analysis for treated subjects in 1.0×10^8, 5.0×10^8, 1.0×10^9 groups. Related to Figure 5.

Indicator	Statistics	1.0×10^8 N=4	5.0×10^8 N=3	1.0×10^9 N=3	Total N=10	P value
OS(Month)	Death	3(75.0)	3(100)	3(100)	9(90.0)	
	Censored	1(25.0)	0	0	1(10.0)	
	Median	14.6	18.3	3.2	8.4	0.107
	95%CI	(0.16, 23.56)	(8.38, 19.84)	(2.56,4.01)	(0.16,19.84)	
PFS(Month)	PD/Death	3(75.0)	3(100)	3(100)	9(90.0)	
	Censored	1(25.0)	0	0	1(10.0)	
	Median	3.39	6.83	1.35	1.48	0.085
	95%CI	(0.16,5.42)	(2.73,12.02)	(1.15,1.48)	(0.16, 6.83)	

Censored：One patient of censored in OS is S006, who was lost to follow-up after being discharged;
One patient of censored in PFS is S010, who underwent subsequent treatment immediately after the initial evaluation for SD.

S7B. DCR results of treated subjects in 1.0×10^8, 5.0×10^8, and 1.0×10^9 dose groups. Related to Figure 5.

Indicator	Statistics	1.0×10^8 N=4	5.0×10^8 N=3	1.0×10^9 N=3	Total N=10
DCR-6W	N(Missing)	3(1)	3(0)	3(0)	9(1)
	DCR(%)	2(50.0)	3(100)	0	5(50.0)
DCR-12W	N(Missing)	1(3)	3(0)	0(3)	4(6)
	DCR(%)	1(25.0)	2(66.7)	0	3(30.0)

Missing：S004 died on day 5 after cell infusion, and no efficacy evaluation results were obtained at weeks 6 and 12; S006, S010, S024, S025, and S029 were withdrawn from the study before the 12-week assessment.

S7C. OS and PFS analysis for treated subjects in Low& Middle-dose, and High-dose groups. Related to Figure 5.

Indicator	Statistics	Low&Middle-dose (1~5E8) N=7	High-dose (1E9) N=3	Total N=10	P Value
OS(Month)	Death	6(85.7)	3(100.0)	9(90.0)	
	Censored	1(14.3)	0	1(10.0)	
	Median	18.3	3.15	8.38	0.035
	95%CI	(0.16,23.56)	(2.56,4.01)	(0.16,19.84)	
PFS(Month)	PD/Death	6(85.7)	3(100.0)	9(90.0)	
	Censored	1(14.3)	0	1(10.0)	
	Median	5.42	1.35	1.48	0.081
	95%CI	(0.16,12.02)	(1.15,1.48)	(0.16, 6.83)	

Censored：One patient of censored in OS is S006, who was lost to follow-up after being discharged;
One patient of censored in PFS is S010, who underwent subsequent treatment immediately after the initial evaluation for SD.

S7D. DCR results of treated subjects in Low&Middle-dose, and High-dose groups. Related to Figure 5.

Indicator	Statistics	Low&Middle-dose (1~5E8) N=7	High-dose (1E9) N=3	Total N=10	P Value
OS(Month)	Death	6(85.7)	3(100.0)	9(90.0)	
	Censored	1(14.3)	0	1(10.0)	
	Median	18.3	3.15	8.38	0.035
	95%CI	(0.16,23.56)	(2.56,4.01)	(0.16,19.84)	
PFS(Month)	PD/Death	6(85.7)	3(100.0)	9(90.0)	
	Censored	1(14.3)	0	1(10.0)	
	Median	5.42	1.35	1.48	0.081
	95%CI	(0.16,12.02)	(1.15,1.48)	(0.16, 6.83)	

Missing：S004 died on day 5 after cell infusion, and no efficacy evaluation results were obtained at weeks 6 and 12; S006, S010, S024, S025, and S029 were withdrawn from the study before the 12-week assessment.

Supplemental Table S8. OS, PFS and DCR analysis for treated subjects. A OS and PFS analysis for treated subjects with B cell normal and B cell low of B cells groups; B. DCR results of treated subjects with B cell normal and B cell low of B cells; C. Additional OS and PFS analysis for treated subjects with B%N50 and B%L50 levels of B cells groups; D. Additional DCR results of treated subjects with B%N50 and B%L50 levels of B cells. Related to Figure 5.

S8A. OS and PFS analysis for treated subjects with B cell normal and B cell low of B cells groups. Related to Figure 5.

Indicator	Statistics	B cell normal N=3	B cell low N=7	Total N=10	P Value
OS(Month)	Death	3(100)	6(85.7)	9(90.0)	
	Censored	0	1(14.3)	1(10.0)	
	Median	18.3	4.01	8.38	0.409
	95%CI	(14.62,19.84)	(0.16,23.56)	(0.16,19.84)	
PFS(Month)	PD/Death	3(100)	6(85.7)	9(90.0)	
	Censored	0	1(14.3)	1(10.0)	
	Median	6.83	1.35	1.48	0.015
	95%CI	(5.42,12.02)	(0.16,2.73)	(0.16, 6.83)	

Censored：One patient of censored in OS is S006, who was lost to follow-up after being discharged;

One patient of censored in PFS is S010, who underwent subsequent treatment immediately after the initial evaluation for SD.

S8B. DCR results of treated subjects with B cell normal and B cell low of B cells. Related to Figure 5.

Indicator	Statistics	B cell normal N=3	B cell low N=7	Total N=10
DCR-6W	N(Missing)	3(0)	6(1)	9(1)
	DCR(%)	3(100)	2(28.6)	5(50.0)
DCR-12W	N(Missing)	3(0)	1(6)	4(6)
	DCR(%)	3(100)	0	3(30.0)

Missing：S004 died on day 5 after cell infusion, and no efficacy evaluation results were obtained at weeks 6 and 12; S006, S010, S024, S025, and S029 were withdrawn from the study before the 12-week assessment.

S8C. Additional OS and PFS analysis for treated subjects with B%N50 and B%L50 levels of B cells groups. Related to Figure 5.

Indicator	Statistics	B%N50 N=7	B%L50 N=3	Total N=10	P Value
OS(Month)	Death	7(100.0)	2(66.7)	9(90.0)	
	Censored	0	1(33.3)	1(10.0)	
	Median	14.62	3.15	8.38	0.031
	95%CI	(2.56,19.84)	(0.16,3.15)	(0.16,19.84)	
PFS(Month)	PD/Death	6(85.7)	3(100.0)	9(90.0)	
	Censored	1(14.3)	0	1(10.0)	
	Median	5.42	1.35	1.48	0.056
	95%CI	(1.15,12.02)	(0.16,1.48)	(0.16, 6.83)	

B% N50 : Baseline B-cell proportion ≥50% LLN；B% L50 : Baseline B-cell proportion ＜50%LLN
Censored：One patient of censored in OS is S006, who was lost to follow-up after being discharged;
One patient of censored in PFS is S010, who underwent subsequent treatment immediately after the initial evaluation for SD.

S8D. Additional DCR results of treated subjects with B%N50 and B%L50 levels of B cells. Related to Figure 5.

Indicator	Statistics	B%N50 N=7	B%L50 N=3	Total N=10
DCR-6W	N(Missing)	7(0)	2(1)	9(1)
	DCR(%)	5(71.4)	0	5(50.0)
DCR-12W	N(Missing)	4(3)	0(3)	4(6)
	DCR(%)	3(42.9)	0	3(30.0)

B% N50 : Baseline B-cell proportion ≥50% LLN；B% L50 : Baseline B-cell proportion ＜50%LLN
Missing：S004 died on day 5 after cell infusion, and no efficacy evaluation results were obtained at weeks 6 and 12; S006, S010, S024, S025, and S029 were withdrawn from the study before the 12-week assessment.

Supplemental Table S9. A. OS , PFS and DCR analysis for combined factor of B%50 & 1-5 x 10^8 and control dose groups. A. OS and PFS analysis for combined factor of B%50 & 1-5 x 10^8 and control dose groups; B. DCR analysis for combined factor of B%50 &1-5x10^8 dose groups Related to Figure 5.

S9A. OS and PFS analysis for combined factor of B%50 & 1-5 x 10^8 and control dose groups, Related to Figure 5.

Indicator	Statistics	B%50&1~5E8 N=5	Control N=5	Total N=10	P Value
OS(Month)	Death	5(100)	4(80.0)	9(90.0)	0.003
	Censored	0	1(20.0)	1(10.0)	
	Median	18.3	3.15	8.38	
	95%CI	(8.38,23.56)	(0.16,4.01)	(0.16,19.84)	
PFS(Month)	PD/Death	4(80.0)	5(100)	9(90.0)	0.003
	Censored	1(20.0)	0	1(10.0)	
	Median	6.13	1.35	1.48	
	95%CI	(2.73,12.02)	(0.16,1.48)	(0.16, 6.83)	

B%50&1~5x 10^8: Baseline B-cell proportion ≥50% LLN with dose of 1~5x10^8
Censored：One patient of censored in OS is S006, who was lost to follow-up after being discharged; One patient of censored in PFS is S010, who underwent subsequent treatment immediately after the initial evaluation for SD.

S9B. DCR analysis for combined factor of B%50 &1-5x10^8 dose groups. Related to Figure 5.

Indicator	Statistics	B%50&1~5x 10^8 N=5	Control N=5	Total N=10
DCR-6W	N(Missing)	5(0)	4(1)	9(1)
	DCR(%)	5(100)	0	5(50.0)
DCR-12W	N(Missing)	4(1)	0(5)	4(6)
	DCR(%)	3(60.0)	0	3(30.0)

B%50&1~5x10^8: Baseline B-cell proportion ≥50% LLN with dose of 1~5x10^8
Missing：S004 died on day 5 after cell infusion, and no efficacy evaluation results were obtained at weeks 6 and 12; S006, S010, S024, S025, and S029 were withdrawn from the study before the 12-week assessment.

Supplemental Table S10. Proportion of $CD19^+$ B cells (%) to leukocytes at Baseline, D7, D14, D28, D42 and D84. Related to Figure 4A.

Groups	Patient	Baseline	Days After Infusion				
			D7	D14	D28	D42	D84
1.0×10^8	S003	12.89	5.1	0	0	1	9.7
	S004	2.30	-	-	-	-	-
	S006	3.5	4.2	0.1	0.5	0.5	-
	S010	5.4	2.7	0.3	0.4	1.2	-
5.0×10^8	S014	11	8.5	4.4	4.4	5.2	7.6
	S016	11.1	6.4	4.6	4.9	4.7	7.1
	S023	7.5	6.9	0.3	0.6	1.4	7.8
1.0×10^9	S024	8.4	0.6	2.5	2.7	0.8	-
	S025	6	0.1	0	0.4	0.4	-
	S029	2	-	0	0.3	0.1	-
TOTAL	N(Missing)	10(0)	8(2)	8(2)	8(2)	8(2)	4(6)
	Mean	7.01	4.31	1.36	1.58	1.70	8.05
	SD	3.84	3.01	1.95	1.91	1.89	1.14
	Median	6.75	4.65	0.30	0.50	1	7.70
	MAX	12.89	8.50	4.60	4.90	5.20	9.70
	MIN	2	0.10	0	0	0.10	7.10

Supplemental Figure S2. Kaplan-Meier analysis of the progression-free survival and overall survival between different combination groups. A. Kaplan-Meier analysis of the progression-free survival and overall survival between different dosage groups; B. Survival analysis for subjects in combined dose groups; C. Survival analysis for subjects in different baseline B%; D. Survival analysis for subjects in baseline B%≥ or < 50%LLN.

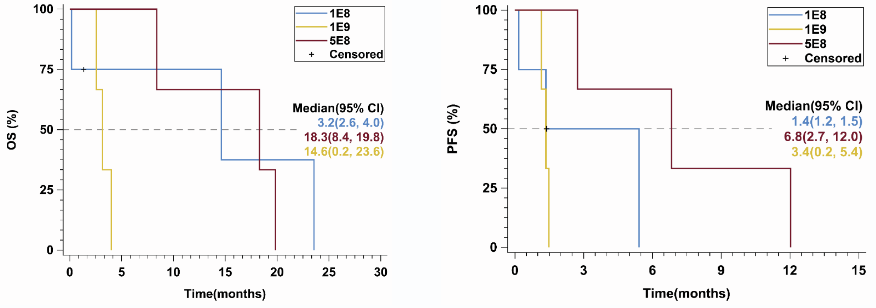

S2A. Kaplan-Meier analysis of the progression-free survival and overall survival between different groups. Left Panel: Overall Survival, OS. Right Panel: Progression-free Survival, PFS. Related to Fig. 5D.

Left Plot (Overall Survival, OS): Lines: Represent survival curves for subjects in different dose groups. - Blue line: Dose group of 1E8. Median OS [95% Confidence Interval (CI)] is 3.2 months (2.6, 4.0). - Yellow line: Dose group of 1E9. Median OS [95% CI] is 18.3 months (8.4, 19.8). - Red line: Dose group of 5E8. Median OS [95% CI] is 14.6 months (0.2, 23.6).
"+" Markers: Indicate censored data points (subjects who did not experience the event of interest, e.g., death, during the study follow - up).

Right Plot (Progression - Free Survival, PFS): - Lines: Represent progression - free survival curves for subjects in different dose groups. - Blue line: Dose group of 1E8. Median PFS [95% CI] is 1.4 months (1.2, 1.5). - Yellow line: Dose group of 1E9. Median PFS [95% CI] is 6.8 months (2.7, 12.0). - Red line: Dose group of 5E8. Median PFS [95% CI] is 3.4 months (0.2, 5.4).
"+" Markers: Indicate censored data points (subjects who did not have disease progression during the study follow - up).

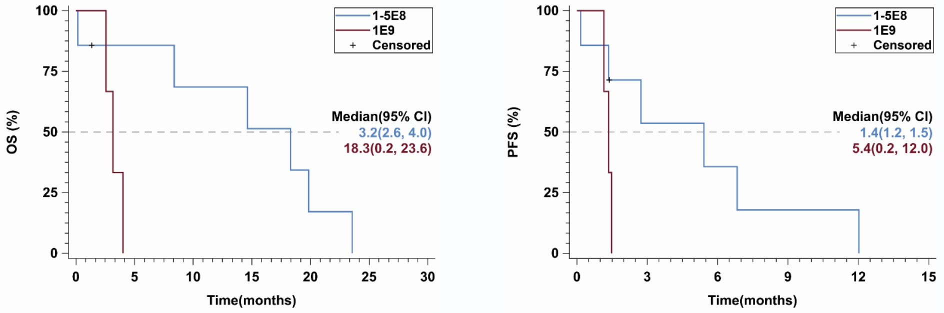

S2B Survival analysis for subjects in combined dose groups. Left Plot: Overall Survival, OS. Right Plot: Progression-Free Survival, PFS. Related to Fig 5D.

Left Plot (Overall Survival, OS) : Lines: - Blue line: Combined dose group "1 – 5x10^8". Median OS [95% Confidence Interval (CI)] is 3.2 months (2.6, 4.0). - Red line: Dose group "1x10^9". Median OS [95% CI] is 18.3 months (0.2, 23.6).

Right Plot (Progression - Free Survival, PFS): Lines: Blue line: Combined dose group "1 – 5x10^8". Median PFS [95% CI] is 1.4 months (1.2, 1.5). Red line: Dose group "1x10^9". Median PFS [95% CI] is 5.4 months (0.2, 12.0).

"+" Markers: Indicate censored data (subjects without disease progression during follow - up).

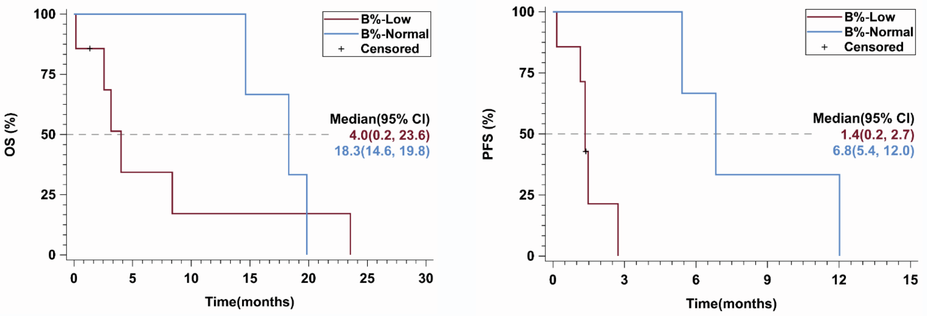

S2C Survival analysis for subjects in different baseline B%. Left Plot: Overall Survival, OS. Right Plot: Progression-Free Survival, PFS. Related to Figure 5D

Left Plot (Overall Survival, OS): Lines: Red line: Group with low baseline B% (B%-Low). Median OS [95% Confidence Interval (CI)] is 4.0 months (0.2, 23.6). Blue line: Group with normal baseline B% (B%-Normal). Median OS [95% CI] is 18.3 months (14.6, 19.8).

Right Plot (Progression-Free Survival, PFS): Lines: Red line: Group with low baseline B% (B%-Low). Median PFS [95% CI] is 1.4 months (0.2, 2.7). Blue line: Group with normal baseline B% (B%-Normal). Median PFS [95% CI] is 6.8 months (5.4, 12.0).

"+" Markers: Indicate censored data points (subjects who did not have disease progression during the study follow-up

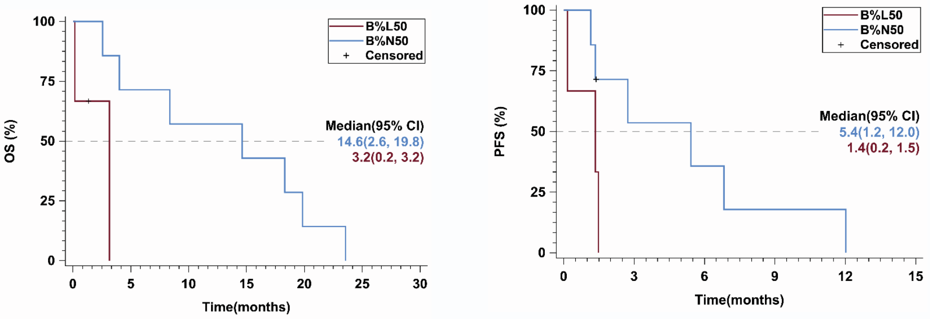

S2D Survival analysis for subjects in baseline B%≥ or < 50%LLN. Left Plot: Overall Survival, OS. Right Plot; Progression-Free Survival, PFS. Related Figure 5D.

Left Plot (Overall Survival, OS): Lines: Red line: Group with baseline B% < 50% of the lower limit of normal (B%L50). Median OS [95% Confidence Interval (CI)] is 3.2 months (0.2, 3.2). -Blue line: Group with baseline B% ≥ 50% of the lower limit of normal (B%N50). Median OS [95% CI] is 14.6 months (2.6, 19.8).

Right Plot (Progression-Free Survival, PFS) : Lines: Red line: Group with baseline B% < 50% of the lower limit of normal (B%L50). Median PFS [95% CI] is 1.4 months (0.2, 1.5). Blue line: Group with baseline B% ≥ 50% of the lower limit of normal (B%N50). Median PFS [95% CI] is 5.4 months (1.2, 12.0).

"+" Markers: Indicate censored data points (subjects who did not have disease progression during the study follow-up).

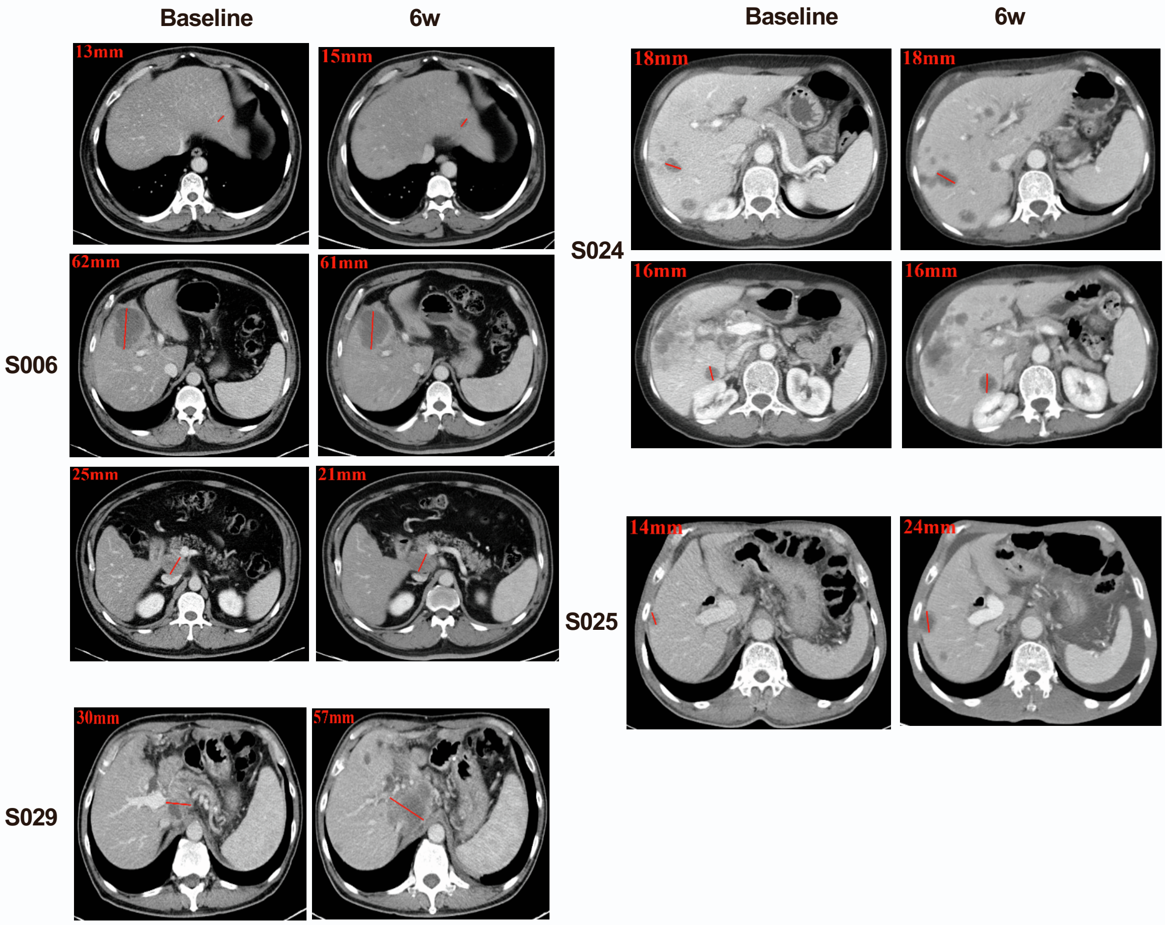

Supplemental Figure S3. Target lesions' CT imaging of baseline and different evaluation time points in progressive disease patients. CT images of patients (S006, S024, S025, S029) with low baseline B cells at baseline and 6 weeks post-treatment are presented. **Related Figure 5A.**

Computed tomography (CT) images of patients (S006, S024, S025, S029) with low baseline B cells at baseline and 6 weeks (6w) post-treatment are presented. For S006, one lymph node (upper panel) and two liver lesions (middle and lower panel) are shown; for S024, two liver lesions; for S025, one liver lesion; and for S029, one lymph node, all at baseline and 6 weeks post-treatment. Lesion sizes (in milli-meter) are indicated in red, and red arrows highlight target lesions. S006 and S024 showed stable disease with no significant change in lesion size. In contrast, S025 and S029 showed an increase in lesion size at 6 weeks compared to baseline.

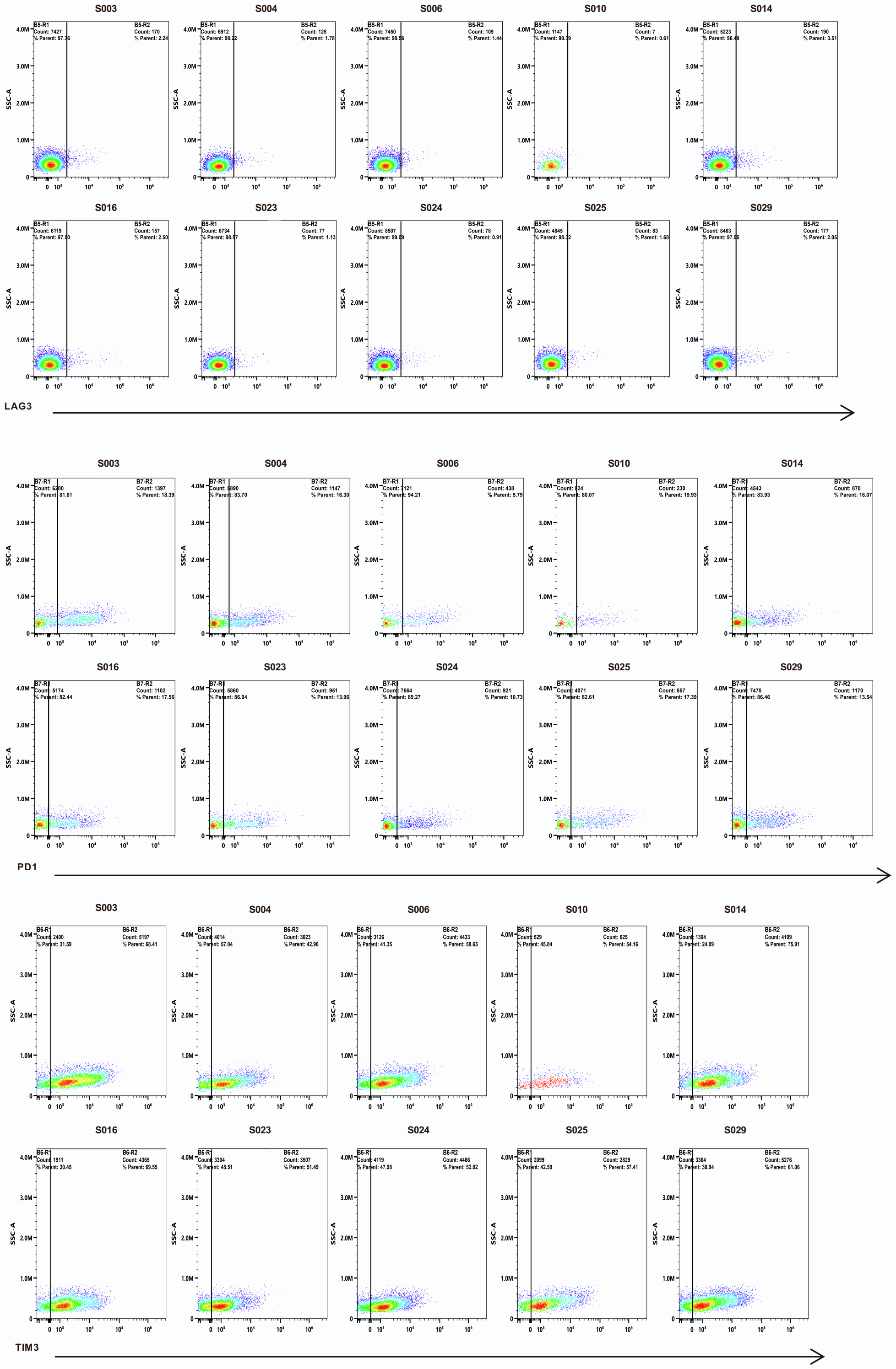

Supplemental Figure S4. Analysis of percentages of PD1, LAG3 and TIM3T in cell products from different patients

T cell products from different patients are stained with commercial antibodies against PD1, LAG3 and TIM3 respectively and analysed with flow cytometry. Related Figure 4.

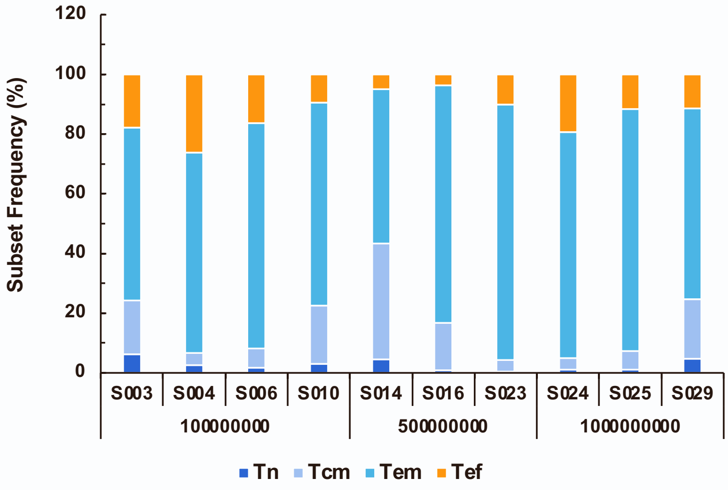

Supplemental Figure S5. Memory phenotypes of different dosages of T cell products from different patients. Related Figure 4 and 5.

Memory phenotypes are characterized by surface staining of CD45RA and CD62L and analysed with flow cytometry (Tn: CD45RA+/ CD62L+; Tcm: CD45RA-/ CD62L+; Tem: CD45RA-/ CD62L- and Tef: CD45RA+/ CD62L-).
